# Growth produces coordination trade-offs in *Trichoplax adhaerens*, an animal lacking a central nervous system

**DOI:** 10.1073/pnas.2206163120

**Published:** 2023-03-10

**Authors:** Mircea R. Davidescu, Pawel Romanczuk, Thomas Gregor, Iain D. Couzin

**Affiliations:** ^a^Department of Ecology and Evolutionary Biology, Princeton University, Princeton, NJ 08544; ^b^Department of Biology, Institute for Theoretical Biology, Humboldt Universität zu Berlin, Berlin D-10099, Germany; ^c^Bernstein Center for Computational Neuroscience, Berlin D-10115, Germany; ^d^Science of Intelligence, Research Cluster of Excellence, Berlin D-10587, Germany; ^e^Lewis-Sigler Institute for Integrative Genomics, Joseph Henry Laboratories of Physics, Princeton University, Princeton, NJ 08544; ^f^Department of Developmental and Stem Cell Biology, CNRS UMR3738, Institut Pasteur, Paris 75015, France; ^g^Department of Collective Behaviour, Max Planck Institute for Animal Behavior, Konstanz 78464, Germany; ^h^Department of Biology, Chair of Biodiversity and Collective Behaviour, University of Konstanz, Konstanz 78464, Germany; ^i^Centre for the Advanced Study of Collective Behavior, University of Konstanz, Konstanz 78464, Germany

**Keywords:** multicellularity, collective, behavior, biophysics, simulation

## Abstract

How collectives remain coordinated as they grow in size is a fundamental challenge affecting systems ranging from biofilms to governments. This challenge is particularly apparent in multicellular organisms, where coordination among a vast number of cells is vital for coherent animal behavior. However, the earliest multicellular organisms were decentralized, with indeterminate sizes and morphologies, as exemplified by *Trichoplax adhaerens*, arguably the earliest-diverged and simplest motile animal. We investigated coordination among cells in *T. adhaerens* by observing the degree of collective order in locomotion across animals of differing sizes and found that larger individuals exhibit increasingly disordered locomotion. We reproduced this effect of size on order through a simulation model of active elastic cellular sheets and demonstrate that this relationship is best recapitulated across all body sizes when the simulation parameters are tuned to a critical point in the parameter space. We quantify the trade-off between increasing size and coordination in a multicellular animal with a decentralized anatomy that shows evidence of criticality and hypothesize as to the implications of this on the evolution hierarchical structures such as nervous systems in larger organisms.

## Significance

Coordination between cells is fundamental for multicellular life. The first multicellular animals were capable of growing to indeterminate sizes but lacked nervous systems that could facilitate coordination. We investigate how size variation affects coordination in such organisms by measuring the collective order in the locomotion of *Trichoplax adhaerens*, the simplest multicellular animal. We find that collective order decreases as such animals grow in size and use a simulation model to determine that this scaling phenomenon occurs at the phase transition between ordered and disordered movement, also known as criticality. Our findings therefore suggest a fundamental trade-off between increasing size and coordination in such a decentralized organism and provide evidence for the necessity of centralized control at larger sizes.

Coordination in collective systems is an area of active research in disciplines as diverse as biophysics, ecology, and engineering (1–4). Multicellular organisms, in particular, require coordination among their constituent cells in order to execute coherent behaviors. However, the earliest multicellular organisms were decentralized (5) and frequently were of indeterminate size and morphology (6). Understanding whether and how such decentralized body plans produce coordinated behaviors is thus essential for understanding the evolution of multicellularity, especially as the earliest diverged animals lacked nervous systems (7–9).

**Fig. 1.**
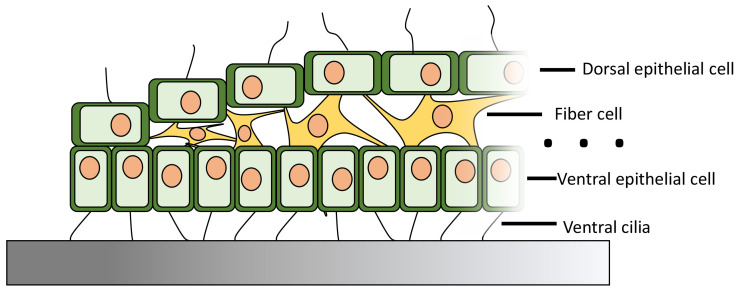
Partial cross-section of *T. adhaerens*. The animal consists of three layers of cells: a dorsal and ventral epithelium layer, with an inner layer of fiber cells. The dorsal and ventral cells are ciliated, with the ventral cilia providing propulsive force for crawling along surfaces, the animal’s primary form of locomotion. Not shown are other specialized cells, such as digestive and crystal cells, that are not directly responsible for movement (for full anatomical details see ref. (13)).

*Trichoplax adhaerens*, arguably the simplest multicellular animal (10, 11) found in the very early diverged Placozoa phylum, faces such a coordination challenge. *T. adhaerens* is a disk-shaped or amoeboid marine organism that can range in size from a hundred micrometers to several millimeters, though it is only 20 micrometers thick (Fig. 1). It is composed of three horizontally stacked layers of tissue consisting of six cell types, including an upper and lower layer of ciliated cells and a middle layer of “fiber” cells that could function as a connective or possibly even a protomuscular structure (12). The animal has no axis of symmetry, no neurons, and crawls on surfaces through the collective action of thousands of cilia on the ventral layer that can beat in any direction (13). How *T. adhaerens* is able to coordinate the beating direction of tens of thousands of ciliated cells in the absence of bilateral symmetry, cephalization, or any other anatomical traits that would be advantageous for coordinated locomotion is an interesting biological phenomenon (14). It is notable that the motile Placozoa are considerably smaller than other animals with decentralized anatomy, such as the Porifera, and that those larger animals tend to be sessile except in their smaller larval stages (15). This correlation between larger size and being sessile has prompted the hypothesis that decentralization is detrimental for coordination in larger organisms (6), but this hypothesis has never been rigorously tested.

Here, we investigate how coordination of locomotion changes in *Trichoplax adhaerens* at different sizes. We measure the collective order of cellular movement and quantify correlations in local fluctuations of this movement, analyzing animals varying in size by nearly an order of magnitude. Through this, we were able to determine the limits in size at which such decentralized control can produce and maintain collective order.

## 1. Results

**Fig. 2.**
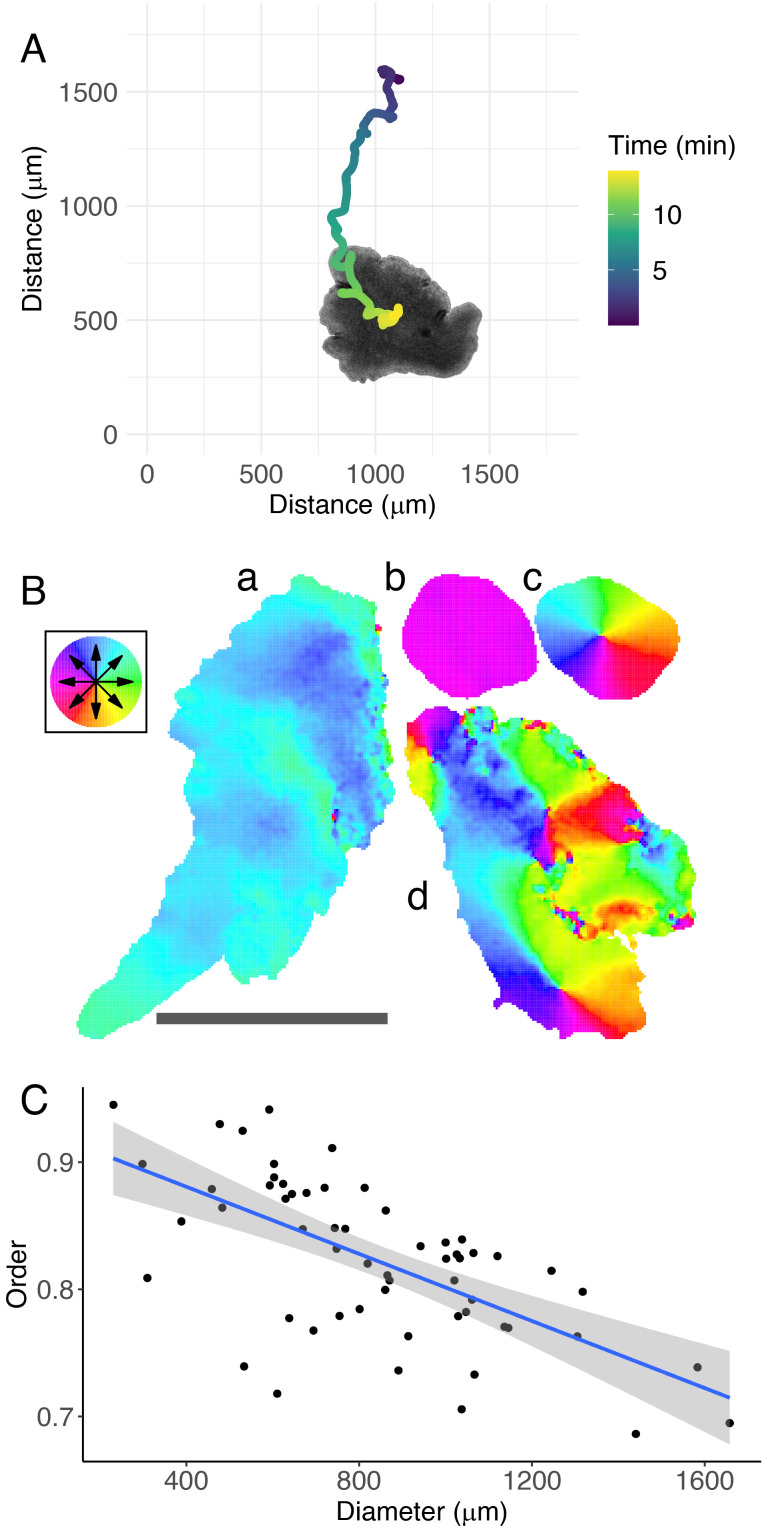
Measuring collective order in the tissue dynamics of *T. adhaerens*. (*A*) A *T. adhaerens* individual with the previous 14 min of its trajectory shown as an overlay. (*B*) Representative snapshots of the optical flow field measured for *T. adhaerens* individuals in different collective states of locomotion. This includes large (*A*) and small (*B*) animals in polarized locomotion, a small rotating animal (*C*), and a large disordered animal (*D*). (*C*) Mean collective order is inversely correlated with individual size, each point indicating the mean diameter and mean collective order for each individual. Blue line: Linear regression of collective order on animal diameter, with the shaded area representing the 95% CI (adj. *R*^2^ = 0.40, *P* <  5.81 × 10^−8^). Error bars (too small to be visible) represent SE based on 500 evaluated frames for each animal.

The ciliated cells of *T. adhaerens*, each of which is able to beat in variable directions (13), allows the animal to traverse its environment at around 5 μm per second. We developed a high-resolution automated tracking microscopy system that allows us to observe the ventral surface of *T. adhaerens* individuals during locomotion, being able to record both the displacement of the individual animal and the relative movement of constitutive tissues within the animal (Fig. 2*A*). We observed the intercellular dynamics involved in locomotion for 58 animals varying in linear size by nearly an order of magnitude, from *c.* 200 µm to 2000 µm in diameter, quantifying the internal deformations using optical flow (Fig. 2*B*). For *T. adhaerens*, the thin body plan of the animal produces a granular texture on the ventral epithelium when illuminated from above, marking the cells of the tissue. The ventral ciliated cells of *T. adhaerens* have a diameter of *c.* 2 micrometers while our recordings are roughly at *c.* 1.2 micrometers per pixel. While this is sufficient to visualize individual cells, it does not allow for the direct observation of cilia. We therefore used optical flow to track the tissue movement by subdividing this texture into regions and tracking the texture movement within each region. We chose the size of these regions to approximate the size of an individual fiber cell diameter or a 10 × 10 cluster of ciliated cells.

Individual animals exhibited a variety of behavioral states throughout the period of recording, ranging from highly ordered (polarized) locomotion, to highly ordered rotating motion, to disordered movement. We quantify the degree of collective order in the resultant vector fields through order parameters that represent polarization (Eq. **1**), rotation/vorticity (Eq. **2**), and expansion/contraction (Eq. **3**) (equations from ref. (16) and (17)). In these equations, *N* is the number of regions/cells that we are tracking, ${\hat{r}}_{\mathrm{ic}}$ is the unit vector from the animal’s center of mass to the region, and $\hat{v_{i}}$ is the unit vector of the region’s velocity. We thereby produced the instantaneous collective order measures that represented the movement of the animal’s cells for each half-second of locomotion.

[1] $$\begin{array}{c} P\left(t\right)=\frac{1}{N}\left|\sum_{i=1}^{N}{\hat{v}}_{i}\right|. \end{array}$$

[2] $$\begin{array}{c} R\left(t\right)=\frac{1}{N}\left|\sum_{i=1}^{N}{\hat{r}}_{\mathrm{ic}}\times {\hat{v}}_{i}\right|. \end{array}$$

[3] $$\begin{array}{c} \Delta \left(t\right)=\frac{1}{N}\sum_{i=1}^{N}∥{\hat{r}}_{\mathrm{ic}}\cdot {\hat{v}}_{i}∥. \end{array}$$

*T. adhaerens* dynamically moves through different states of collective movement throughout its locomotion, shifting from polarized, to rotating, to disordered movement. We therefore represented the overall collective order exhibited by an animal through a unified order measure $O\left(t\right)=\sqrt{P{\left(t\right)}^{2}+R{\left(t\right)}^{2}+\Delta {\left(t\right)}^{2}}$, which we refer to as the collective order. This value is bound from 0, which represents completely disordered motion, to 1, which represents a pure affine transformation. By considering the mean collective order exhibited by an animal over time, we find that larger animals are significantly less ordered than smaller ones (Fig. 2*F*; linear fit: t = −5.73, p <  5 × 10^−7^; adj.R^2^ = 0.30). This suggests that larger animals have greater difficulty establishing a consensus of locomotion across their body plans, resulting in the animal’s characteristic shape changes (18).

This observed decrease in collective order with animal size poses an important question: Is this trade-off between size and collective order inevitable in such decentralized animals, or can it be avoided under the right conditions? For instance, larger *T. adhaerens* individuals may become disordered because the coupling interactions between cells (mechanical or otherwise) may be insufficiently strong to propagate movement information beyond a certain spatial range, causing cells on one side of the animal to be unaware of changes in movement on the other. Strengthening these interactions could make it easier to maintain collective order, but would such a change make the animal unresponsive to perturbations and new information from the environment?

**Fig. 3.**
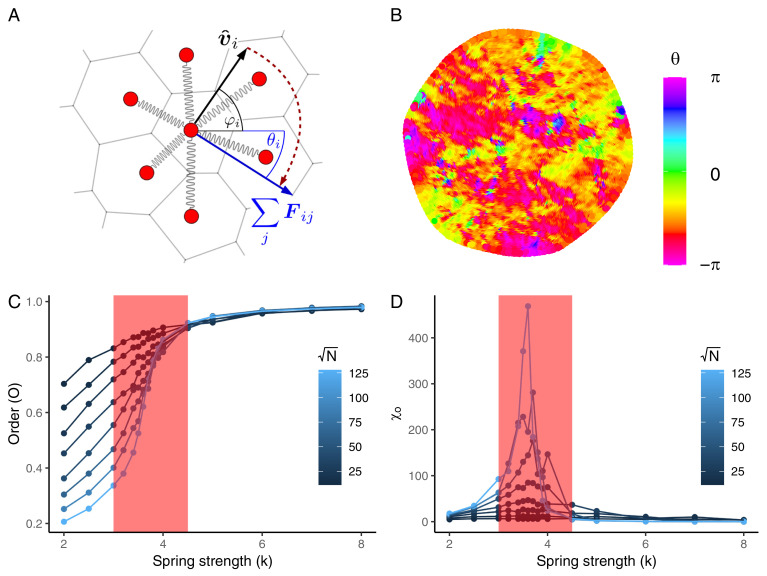
Simulated cellular sheets in a critical regime replicate the relationship between size and order observed in *T. adhaerens*. (*A*) Schematic of the elastic SPP simulation, consisting of particles that influence the direction of movement of their Voronoi neighbors using spring-like interactions. Here, $\hat{v_{i}}$ represents the instantaneous velocity of particle *i*, *θ*_*i*_ represents the direction of motion of particle *i*, and *F*_*ij*_ represents the elastic force that particle *j* exerts on *i*, which will influence both ${\hat{v}}_{i}$ and *θ*_*i*_. (*B*) A representative snapshot of a simulation consisting of *c.* 16,000 particles. Color represents the heading *θ*_*i*_ of individual particles. (*C*) Mean collective order observed in simulations of varying size as a function of the coupling strength *k*. The region highlighted in red represents the location of the second-order phase transition. (*D*) Susceptibility *χ*_0_ of systems of different sizes in relation to the interparticle coupling strength *k*. The divergent *χ*_0_ in the red region indicates a critical regime.

In order to explore these possibilities, we developed a simple yet generic model of *T. adhaerens* as a distributed, collective system, where we can systematically tune model parameters to identify the regime that best recapitulates our experimental observations. Our simulated representation consists of a mesh of self-propelled particles (SPPs) with elastic interactions (*Methods* and Fig. 3*A*). Similar elastic interaction models have been used previously to simulate *T. adhaerens* locomotion (13), though these prior models relied on an alignment interaction rule to generate coordinated locomotion. However, it is unclear whether and how individual cells can sense orientations of neighboring cells, which is implicitly assumed by an explicit alignment interaction. We also note that the cilia-driven propulsion of Placozoa takes place on a substrate, which is from a physics point very different from collective ciliary swimming like in *Volvox carteri* (8) since the substrate greatly reduces the importance of long-range hydrodynamical couplings (19). We therefore developed a model—inspired by another model for collective cell migration (20)—that is able to generate order even in the absence of such a rule. In our model, each particle uses only information provided by elastic forces— whose strength is governed by a spring constant *k*—and coordination emerges from coupling of the self-propelled direction of the particles to the elastic force they experience (see also ref. (21) and (22) for related models). Our model is similar to another recently produced model of Placozoa tissue dynamics (23), though while this other model more explicitly accounts for cilia oscillations, their analysis is performed in the limit of weak coupling between the propulsion orientation and cilia oscillations. It therefore provides a detailed discussion for the emergence of long-ranged correlations in the ordered state (see also ref. (22)) but did not fully explore the parameter space that results in the transition from ordered or disordered movement.

It was previously demonstrated in other SPP models that such collective systems can undergo a phase transition from disordered to ordered locomotion by tuning a variety of parameters governing interparticle interactions (16, 24). In our model, we show that by varying only the spring constant *k*, and keeping all other parameters constant, it is possible to control the degree of order in the simulated animals, and thereby cause the animal to transition from a disordered to ordered locomotion (Fig. 3*C*), with the continuous phase transition occurring near the critical point *k*_*c*_ ≈ 3.7 (red highlight). We confirm this is the critical point by measuring the susceptibility *χ*_*o*_ of the system as in ref. (25) (Fig. 3*D*), a parameter which is known to diverge (maximize) at criticality (26–28). Our results also recapitulate a well-known phenomenon of finite-size systems, namely that the control parameter value that maximizes susceptibility changes with animal size.

The phase transition in our system reveals several relationships between the interaction strength *k*, system size *N*, and collective order *O*. First, collective order decreases with system size only in the critical and the disordered regimes. Second, collective order decreases more quickly in response to weakening *k* for larger system sizes. Thirdly, the narrow range of collective order we observe in the actual *T. adhaerens* (Fig. 2*C*) is reproduced only in simulations in the critical regime (red highlight). These combined observations suggest that *T. adhaerens* exists in this special parameter region, which could place Placozoa in a family of other biological collective systems that have been used as evidence for the criticality hypothesis (29).

**Fig. 4.**
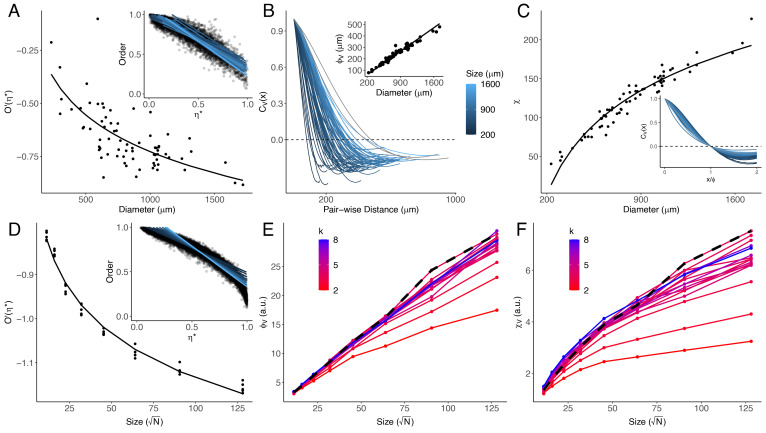
Statistical properties of *T. adhaerens* tissue dynamics are reproduced by simulations tuned to criticality. (*A*) The slope of the relationship between collective order and fluctuation energy with the black line representing the best fit logarithmic model approximating the data. *Inset*: The relation between the proxy intrinsic noise η^*^ and the instantaneous collective order. Lines represent linear regressions for individual animals, with brighter blue lines indicating larger sizes. (The color scale is the same as in panel *B*). The slopes of these regressions provide the values for *O*′(η^*^) in the main panel. (*B*) Velocity correlation (*C*_*V*_(*x*)) profiles for animals of different sizes, with the pair-wise distance representing intercellular distances within an animal. The color scale represents animal size (equivalent diameter). *Inset*: The mean correlation length (*C*_*V*_(*x*)=0 = *λ*_*v*_) plotted against animal diameter for animals of varying sizes, showing a strong linear relationship. (*C*) The relationship between correlation integral (*χ*) and animal size. Individual animal means are shown as data points. The best-approximating sublinear regression line is shown ($\chi =\alpha D^{\beta }+\gamma ;\alpha =5.46;\beta =0.524;\gamma =-60.9$). *Inset*: Correlation profiles of animals with pair-wise intercellular distances normalized by the animal’s respective correlation length *λ*_*v*_. Color scales are the same as in (*B*). (*D*) The slope of the relationship between collective order and fluctuation energy in simulated systems near criticality (3.0 ≤ *k* ≤ 4.0), with the best logarithmic model fit shown in black. Inset: The relation between fluctuation energy fraction and instantaneous collective order. Lines represent linear fits for individual simulations, with brighter blue lines representing larger systems. The slopes of these regressions provide the values for *O*′(η^*^) in the main panel. The simulated system in the critical regime shows the same relationships as observed in actual animals (*A*). (*E*) The velocity correlation length, *θ*_*v*_, for simulated systems of varying size, with color representing the spring coupling constant *k*. The line corresponding to the critical regime (*k* = 3.5) is highlighted in black, showing that the linear relationship between system size and correlation length is present in this regime, showing the same relationship as exhibited by actual animals (*B* *inset*) (*F*) The integral of the correlation profiles, also known as the susceptibility *χ*_*v*_, for simulated systems of varying size, with color representing the spring coupling constant *k*. As in (*E*), the black dashed line represents a system in the critical regime, showing the same relationship as exhibited by actual animals (*C*).

Determining exactly which simulated regime best represents *T. adhaerens* requires investigating several other known statistical properties and phenomena. First, we investigated the rate at which collective order decreases as we change our control parameter *k*, as it is known that this phase transition will be steeper in larger collective systems (24, 30). Our simulations reveal this pattern as well in the critical regime (Fig. 3*C*), but while it is trivial to adjust the control parameter *k* in our simulations, we cannot perform a similar manipulation in *T. adhaerens*. However, we can assume that corresponding control parameters in *T. adhaerens* must undergo some natural dynamic variation due to various noise sources intrinsic to living systems. We therefore define a proxy noise value $\eta^{*}=\frac{\left\langle \|u^{2}\|\right\rangle }{\left\langle \|v^{2}\|\right\rangle }$, where **v** represents the full velocity vector of a given particle or cell, and **u** represents the velocity fluctuation vector, which is the velocity that remains once the collective movement components have been subtracted (*Materials and Methods*). Where noise is maximal (η^*^ = 1), the velocity fluctuation represents the entirety of the cell velocity, and there is no collective movement component. This quantity can be measured both in *T. adhaerens* and our simulations and is found to be strongly inversely correlated with the control parameter *k* (*SI Appendix*). By measuring the instantaneous η^*^ and *O* at multiple time points for animals (Fig. 4 *A*, *inset*) and simulations (Fig. 4*D*, *inset*) of varying sizes, we are able to characterize the steepness of the phase transition, defined as *O*′(η^*^), and show that it is steeper (more negative) with increasing organism size in both *T. adhaerens* (Fig. 4*A*) and simulations (Fig. 4*D*).

Another important measure of coordination in collective movement is the spatial correlation of the velocity fluctuations between individual cells/particles, a method proposed by Cavagna et al. (31) that indicates the range at which cells influence each other’s movement. In this measure (Eq. **4**), *i* and *j* are two particles or cells separated by a distance *r*_*ij*_, and their respective velocity fluctuations are *u*_*i*_ and *u*_*j*_. We normalize the correlations at all pair-wise distances by a constant *c*_*o*_ such that *C*(0)=1, with the range bound between 1 (perfect correlation) and −1 (perfect anticorrelation). We use the summations and Kronecker delta function *δ*(*r* − *r*_*ij*_) to determine the mean correlation for any given distance *r* and thereby characterize the correlation function *C*(*r*) across the entire animal body (Fig. 4*B*).

[4] $$\begin{array}{c} C\left(r\right)=\frac{1}{c_{o}}\frac{\sum_{\mathrm{ij}}u_{i}u_{j}\delta \left(r-r_{\mathrm{ij}}\right)}{\sum_{\mathrm{ij}}\delta \left(r-r_{\mathrm{ij}}\right)}. \end{array}$$

We characterized the observed correlations by the velocity correlation length *λ*_*v*_, with *C*(*λ*_*v*_)=0. The general expectation for interacting particle systems with local coupling is that *λ*_*v*_ has a finite, constant value that is independent of system size and determined by the relative strength of the coupling between particles. However, we find that *λ*_*v*_ scales linearly with animal size (Fig. 4*B*; *β* = 0.26, *p* <  10^−16^, adj.R^2^ = 0.96) across the entire size distribution of *T. adhaerens*, a phenomenon that has been noted in other biological collectives and that has been argued to be an indicator of criticality (17, 31–33). By measuring this quantity in simulated systems, we find that linear scaling occurs both in critical and ordered regimes, while simulations in the disordered regime exhibit sublinear scaling (Fig. 4*E*). This result is consistent with the behavior of simulated elastic sheets of SPPs described in other studies (22).

In addition to the correlation length, we can further describe the correlation profile by its integral *χ* measured from *r* = 0 to *λ*_*v*_ (17, 34). We find that this quantity increases sublinearly with animal diameter in *T. adhaerens* (Fig. 4*C*), which is visually apparent if one rescales each animal’s correlation profile *C*(*r*) by *λ*_*v*_ to produce *C*(*r*/*λ*) (Fig. 4*C*, *inset*). This is in contrast to other biological collectives like bacterial swarms or bird flocks where both *λ*_*v*_ and *χ* increased linearly with size, such that the correlation profiles could be collapsed onto a scale-invariant function (31, 33, 35). By contrast, in *T. adhaerens*, we measure a roughly five-fold increase in *χ* over a corresponding seven-fold increase in linear size, a ratio that we observe for our simulations in the critical regime (*k* ≈ 3.5; Fig. 4*F*, black highlight).

Through our investigation, we were able to characterize the effect of size on coordination in *T. adhaerens* and identify the parameter regime in simulations that best replicated the empirical observations. We find that animals become increasingly disordered in their locomotion as they increase in size, a phenomenon that occurs in our simulations in both the critical and low-order regimes. When we investigated the statistical properties of the velocity fluctuations, we found that *T. adhaerens* exhibits a linear scaling of the correlation length and sublinear scaling of the correlation integral with animal diameter. When compared with our simulations, both of these scaling phenomena are best recapitulated by simulations tuned to either the critical or ordered regimes. The combination of our observations in *T. adhaerens* therefore occur only when the model is tuned to a critical regime, suggesting that a similar tuning may be responsible for the phenomena in the organism.

## 2. Discussion

The capacity of a collective system to coordinate as it increases in size is essential for all decentralized systems, including the first multicellular animals such as *T. adhaerens*. By finding that order decreases as *T. adhaerens* increases in size, we identified a trade-off in early decentralized organisms between growth and coordination. We used simulation modeling to replicate this phenomenon and showed through a variety of statistical observations that only simulations tuned to a critical point can replicate the entirety of our observations in *T. adhaerens*. This work provides support for the so-called “criticality hypothesis” that biological systems are tuned to phase transitions that optimize collective information processing (29, 36), and it was shown that being at this regime of instability enables collective agility and responsiveness (37). Our simulation also indicates that this trade-off between size and collective order—by occurring even at the critical regime—suggests that this coordination challenge is inescapable as such decentralized organisms grow in size. This raises questions and interesting hypotheses for why more complex coordination structures and body plans evolved in later-diverged multicellular animals.

It is interesting to understand the limitations to coordination in *T. adhaerens* by contrasting them with other biological collectives, such as starling flocks (31) or fish schools (32). These animal groups often grow to tremendous scales and yet can still achieve highly ordered and coordinated behaviors. By comparing cells in *T. adhaerens* to animals in groups, we can identify multiple restrictions on the former that place them at a distinct disadvantage in coordinating at scale. One such restriction is that in *T. adhaerens*, the interactions among the constitutive cells are only local, typically in a Voronoi network of nearest neighbors. Comparatively, animal groups can coordinate over long ranges with other sensory modalities such as over a visual network (38, 39). Animals in groups can also quickly switch neighbors at relatively fast timescales compared to cells in *T. adhaerens*, that are physically much more constrained. These additional constraints may explain why such animal groups have scale-invariant correlation profiles that collapse on a single generic function irrespective of size, while in *T. adhaerens*, correlations decay much faster over distance as a proportion of the animal size. These limitations of simple multicellular life may compound each other and explain why large animals lacking a nervous system are typically sessile.

Our study has several limitations that provide promising expansions for future work. One limitation is our simplified representation of *T. adhaerens* as a cellular sheet, abstracting its true but functionally unknown anatomy. In particular, it is unclear whether the internal fiber cells in *T. adhaerens*, whose function and network structure is unknown, is involved in coordination. It was previously considered that these cells are neural or muscular precursors (40), though this was dismissed by more recent anatomical studies (41). however, there is still too little known about their network structure or process lengths to discount them having some role in coordination. In the absence of contradictory evidence, we have assumed that the number of such cells increases in proportion to the animal’s size and that their network topology is largely lattice-like. *T. adhaerens* also possesses specialized cells that bind to neurotransmitter-like molecules (41, 42) and were reported to arrest the beating of nearby cilia during feeding (43). More recent assays have shown that glycine can act as a chemoattractant for *T. adhaerens* (44), which provides a promising avenue for the impact of size on coordination in tracking gradients and contrasting this with similar studies on animal groups (45).

One important implication of the detrimental impact of size on collective order and coordination is that the maximal size of *T. adhaerens* could be limited by an inability to coordinate. It has recently been demonstrated that breakdown in coordinated locomotion in *T. adhaerens* causes microfractures in the animal’s epithelium and that this plays an important role in asexual fission (23). If poor coordination at larger sizes increases the frequency of these microfractures and thereby generates one of the most characteristic behaviors of *T. adhaerens*, it would both reveal this as an emergent phenomenon (as opposed to one that is developmentally or genetically regulated) and provide an interesting parallel to size mediation in animal groups (17, 46).

Our approach extends the knowledge of collective systems to provide insight into the effect of size on coordination in the earliest multicellular animals. We view this as part of a larger effort to reveal not only how system-wide coordination can be achieved spontaneously (47, 48) but also to identify the limitations of such decentralized decision-making. The detrimental effect of size on coordination in such decentralized animals reveals a limitation that such organization poses for coordination and puts forward a hypothesis for the evolution of hierarchical structures as animals were faced with the evolutionary pressure to grow to bigger (49).

### Tracking and Measuring Velocity Fields.

We developed an in-house MATLAB software to segment the animals in each frame, ignoring frames where part of the animal was outside of the camera’s field of view or where the stage was moving. We defined the animal size as the area enclosed within the animal’s two-dimensional footprint, with the animal diameter defined as the diameter of a circle of equivalent area. To minimize measurement error in size caused by out-of-plane buckling of the animal, we considered only frames where the observed area was at least 80% of its maximum observed area. We found no trend of cell size with animal size (*SI Appendix*), in accordance with histological studies (41). We computed the optical flow between frames using software described in ref. (32), keeping vectors within the animal’s footprint, while removing velocity vectors within a distance of 10% of the animal diameter from the boundary to eliminate edge effects on the optical flow algorithm. This leaves a velocity field of instantaneous velocity vectors *v*_*i*_(*t*) for each 100 square micrometer region *i* at all times *t*.

### Calculating Velocity Fluctuations.

For each cell *i*, we use the current position *y*_*i*_(*t*) and current velocity ***v***_*i*_(*t*) to determine the future position *y*_*i*_(*t* + *dt*) of the cell after a time interval *dt*, as defined by the Eq. **5**.

[5] $$\begin{array}{c} y_{i}\left(t+dt\right)=y_{i}\left(t\right)+v_{i}\left(t\right)dt. \end{array}$$

We then define the velocity fluctuation ***u***_*i*_(*t*) as the residual term left after subtracting the optimal affine transformation—defined as the translation **T**, rotation **R**, and dilatation *Δ*—that best transforms all positions *y*(*t*) to *y*(*t* + *dt*), as in Eq. **6**. The optimal values of **T**, **R**, and *Δ* are defined as those that minimize the sum of all of the magnitudes of the fluctuations, $\epsilon \;=\;\sum_{k=1}^{N}∥u_{k}\left(t\right)∥$. In the case of solid-body translation, rotation, or expansion, the movement would be perfectly captured by an affine transformation with the result that *ϵ* = 0. For actual data, the collective movement never matches this ideal, with the remainder terms being the velocity fluctuations.

[6] $$\begin{array}{c} u_{i}\left(t\right)=y_{i}\left(t+dt\right)-T-\Delta Ry_{i}\left(t\right). \end{array}$$

### Correlation Profiles.

In addition to the velocity correlation function defined in Eq. **4**, we also determine the directional correlation *C*_*D*_(*r*) and the speed correlation *C*_*S*_(*r*) defined as

[7] $$\begin{array}{c} C_{D}\left(r\right)=\frac{\sum_{\mathrm{ij}}w_{i}w_{j}\delta \left(r-r_{\mathrm{ij}}\right)}{\sum_{\mathrm{ij}}\delta \left(r-r_{\mathrm{ij}}\right)}. \end{array}$$

[8] $$\begin{array}{c} C_{S}\left(r\right)=\frac{1}{c_{o}}\frac{\sum_{\mathrm{ij}}\eta_{i}\eta_{j}\delta \left(r-r_{\mathrm{ij}}\right)}{\sum_{\mathrm{ij}}\delta \left(r-r_{\mathrm{ij}}\right)}. \end{array}$$

Here, *δ* and *c*_*o*_ have the same meaning as in Eq. **4**. $w_{i}=\frac{u_{i}}{∥u_{i}∥}$ is the unit vector of the velocity fluctuation of cell cluster *i*, and *η*_*i*_ is its speed fluctuation with respect to the global mean speed, $\eta_{i}=∥u_{i}∥-\frac{1}{N}\sum_{k=1}^{N}∥u_{k}∥$.

We measure the slope of the correlation profile at the zero-crossing using the five-point stencil method, fitting a second-order polynomial to the points closest to the crossing and then evaluate the slope as the first derivative of the fitted function at the crossing. We measured the susceptibility (integral) of the correlation profile by applying a cubic spline fit of the average correlation profile, interpolating 100 evenly spaced data points across the domain and then integrating using Simpson’s rule. These quantities in the directional and speed correlations are largely consistent with the full velocity correlations (*SI Appendix*).

### Self-Propelled Particle Model.

The movement *T. adhaerens* as a collective system will be the result of intercellular and intracellular mechanical forces and possibly chemical signaling between neighboring cells. In spite of recent advances in uncovering the detailed structure of *T. adherens* (41), the dynamics of cell coordination remain unknown. In order to systematically study these dynamics, we use an idealized mathematical model, based on three main assumptions:

- Interactions between parts of the animal are local, restricted only to the first shell of neighboring units (Voronoi neighborhood),
- the mechanical interactions can be mapped to an effective “mechanical” spring-like forces, and
- each unit is self-propelled with a preferred direction of motion, which on a finite time scale relaxes toward the average direction of the resulting mechanical force. Furthermore, the heading direction of self-propulsion is subject to fluctuations.

Our model consists of a two-dimensional sheet of *N* coupled self-propelled particles (SPPs), coupled by spring-like interactions on a Voronoi network, which is an effective representation of the simple, two-dimensional anatomy of *T. adhaerens* (13). Each particle corresponds to a “disc” of 20 μm in diameter, the maximum resolved scale that allows us to parameterize our simulations from experimental data. The scale of 20 μm is also the approximate separation of fiber cells within *T. adhaerens*. In our model, the elastic forces represent the network of these fiber cells, believed to provide an elastic scaffolding that spans the entire animal (41) and the elastic cell–cell contact forces caused by intercell adherens junctions and steric volume exclusion. In our model, we account for these mechanisms by an effective elastic force, combining short-ranged repulsion and long-ranged attraction between particles, with an equilibrium distance *r*_0_ between the cell centroids. In addition, we assume also directional noise *η*, which represents fluctuations in the cell’s intrinsic ciliary beating direction. The strength of the elastic interaction forces is controlled via the spring strength *k* relative to *η*. These elastic forces influence the heading direction of individual particles: Each particle adjusts its self-propulsion direction ${\hat{v}}_{i}\left(t\right)$ to align with the sum of mechanical forces exerted by its Voronoi neighbors, with a relaxation time scale *τ*. This results in the self-propelled components aligning their direction of movement with the direction of least mechanical resistance and/or maximal elastic pull.

The displacement of each particle *i* is thus given by an overdamped equation of motion

[9] $$\begin{array}{c} \frac{dr_{i}}{dt}=v_{0}{\hat{v}}_{i}(t)+\mu \sum_{j\in N_{i}}F_{ij}, \end{array}$$

with ***r***_*i*_ being the position vector, *v*_0_ the self-propulsion speed, ${\hat{v}}_{i}$ the unit vector determining the self-propulsion direction, and *μ*-pagination the mobility of the focal particle. Finally, $F_{\mathrm{ji}}=k\;\left(r_{\mathrm{ji}}-r_{0}\right){\hat{r}}_{\mathrm{ij}}$ is a linear, spring-like attraction-repulsion force with the coupling strength *k* and an equilibrium distance *r*_0_, which model the elastic coupling to neighboring particles (*SI Appendix*, *Materials* for details). Without loss of generality, we can set the mobility to *μ* = 1, by rescaling *k* as one of the control parameters, and set *r*_0_ = 1 effectively rescaling all model parameters in terms of the equilibrium distance.

The unit vector of the self-propulsion ${\hat{v}}_{i}={\left(\cos\varphi_{i},\sin\varphi_{i}\right)}^{T}$ is determined by the polar angle *φ*_*i*_, which evolves according to

[10] $$\begin{array}{c} \frac{d\varphi_{i}}{\mathrm{dt}}=\frac{1}{\tau }\sin\left(\theta_{i}-\varphi_{i}\right)+\eta_{i}\left(t\right), \end{array}$$

with *θ*_*i*_ being the polar angle of the total attraction-repulsion force ***f***_*i*_ = ∑_*j*_***f***_*ji*_: *θ*_*i*_ = arctan(*f*_*i*, *y*_/*f*_*i*, *x*_). Thus, *φ*_*i*_ relaxes toward the force direction with a characteristic time *τ*. Finally, *η*_*i*_(*t*) is Gaussian white noise with vanishing correlations: ⟨*η*_*i*_(*t*)*η*_*i*_(*t*′)⟩=2*Dδ*(*t* − *t*′). The noise intensity *σ*. Each particle interacts only with its direct Voronoi neighborhood, and we assume a fixed interaction topology (no neighbor switching). Further model and simulation details are given in *SI Appendix*.

## Supplementary Material

Appendix 01 (PDF)

## Data Availability

Data used for the production of this paper are available on Zenodo at https://zenodo.org/record/6507708.
