# Supplementary material for: Growth produces coordination trade-offs in Trichoplax adhaerens, an animal lacking a central nervous system
Source: Proc Natl Acad Sci U S A. 2023 Mar 10;120(11):e2206163120. doi: 10.1073/pnas.2206163120 (PMC10089153; doi:10.1073/pnas.2206163120)
Supplement: Supplementary file 1 — Appendix 01 (PDF) [file pnas.2206163120.sapp.pdf]

Supporting Information
Growth produces coordination trade-offs in decentralized body plans

Mircea R. Davidescu, Pawel Romanczuk, Thomas Gregor, Iain D. Couzin

I. DIRECTIONAL NOISE AS A TUNABLE CONTROL PARAMETER

In the main text we presented the use of simulations where the spring strength μ was adjusted as a control parameter, demonstrating that we could produce a phase transition in our collective system by tuning this parameter and that only simulations tuned to criticality exhibited all of the properties of *T. adhaerens*. This critical tuning can also be achieved through other control parameters, and here were demonstrate the use of the directional noise η as such a parameter. We find that adjusting the noise in a system can reproduce the same phase transition (Figure 1A). We also recuperate the same scaling of collective order with system size under the critical and disordered regimes. Similarly, the low noise and critical noise regimes show the same scale-free correlations (1B) and sublinear scaling of susceptibility (Figure 1C). Finally, the effect of system size on the steepness of the phase transition is also reproduced by our noise-modulated simulations (Figure 1D). We have therefore demonstrated how - using a completely different control parameter - we are able to tune our simulated system to a critical point and thereby recapitulate the phenomena observed in our biological system.

II. MEASUREMENT SENSITIVITY AND CHARACTERIZATION

A. Fluctuations in *T. adhaerens* compared with noise-induced correlations

Our optical flow algorithm is based on the Horn-Schunk method [1], which while offering very good performance in estimating flow for smooth, continuous motion, will introduce spurious correlations over image discontinuities. To ensure that the range and form of our correlation profiles are not caused by sensitivity to noise, we measured the sensitivity of our optical flow measurement to noise with two controls: a positive control on a scrambled image, and by measuring the flow field produced by an immobile, dead animal, where any movement should be caused by measurement noise of the camera sensor.

We produced white noise video by taking recorded images in our datasets and scrambling the pixels, and then producing a video a from a sequence of such images. Our optical flow algorithm produces long-range correlation artifacts on such a video (Figure 2A), as expected based on the limitations of the algorithm. However, these long-range fluctuations are ephemeral, being completely uncorrelated between subsequent frames (2B). By comparing the fluctuations recorded from real data on animal movement (2C, top) with the fluctuations produced by a scrambled animal image (2C, bottom), we find that fluctuations observed for real data are stable over long time periods, while those produced by noise are completely uncorrelated across time. This very different timescale of the noise-derived fluctuations and the velocity fluctuations from our animal data makes it impossible that our own measurements are artificially-induced by such noise.

A second control we performed was to take a recording of an animal that perished in the middle of our recording and had begun to degenerate, and measure the velocity field within this dead organism. In this case, any correlations that arise should be driven entirely by noise in the camera sensor and illumination system. We performed the same segmentation and fluctuation calculation as in our methods, producing a velocity fluctuation field within the dead animal (Figure 3A). We find that the range of spatial correlations in the velocity fluctuations is significantly smaller than those we record in living animals. When we compare the correlation length of the velocity fluctuations within the dead animal to those recorded in living organisms (3B). Not only is the correlation length for our dead specimen much smaller than what would be expected for an organism of such a given size, but it is also almost half the size as those recorded in even our smallest individuals. We therefore are confident that our measurements are outside the range of those inducible by noise.

B. Measurement error induced by optical flow estimation parameters

The effect of spatial distance on the strength of velocity correlation between cells at different spatial distances is likely to be influenced by the granularity with which we can estimate the local velocity field. Using optical flow, the measurement of this velocity is the result of integrating the movement of a pixel intensity pattern within a

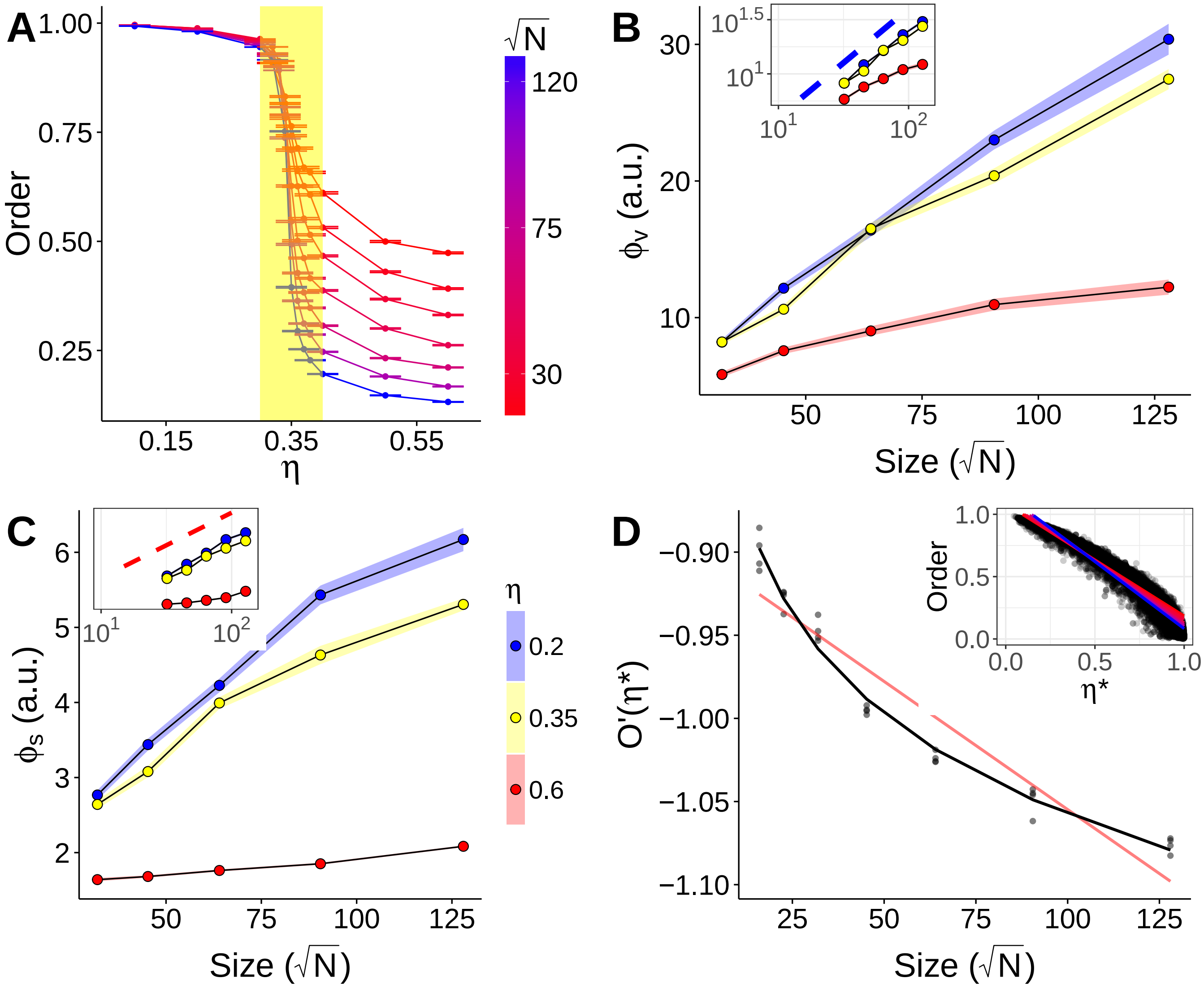

Fig. S 1. **Coordination of tissue dynamics in Placozoa is recapitulated by elastic networks near criticality, using directional noise as the control parameter.** (A) The effect of varying directional noise (η) on collective order for simulated systems of varying size. Red highlight: the phase transition that occurs in the critical range of η_C. Color scale represents the size of the simulated lattice. (B) The relation between system size and the correlation length (ϕ_v), for lattices with sub-critical, critical, and supercritical spring strength parameters. Inset: log-log plot of the same quantities. The dashed blue line represents the empirically-observed linear scaling found in *T. adhaerens*. (C) The relation between systen size and the susceptibility (χ) for systems with different noise levels η. Inset: log-log plot of the same quantities. Dashed lines represent linear (blue), square-root (red), and the scaling exponent of the scaling data. (D) The steepness of the phase transition in collective order as effective noise (η^*) is increased for elastic sheets of different size. Inset: The relation between effective noise (η^*)and collective order for sheets of different size when η is in the critical range between from 0.3 to 0.4.

certain radius of the position of the estimated vector. The granularity with which our optical flow field estimates the movement in a sequence of frames is influenced by two parameters: averaging window size λ and the polynomial expansion n; more details on these parameters are available at [2]. Increasing these values results in optical flow fields that are more robust to image noise and allow for detecting faster motion, but results in a smoother motion field. Our reported results are generated with parameter settings $\lambda = 45$ and $n = 7$; the rest of the parameters are the defaults specified at [3].

We tested the effect of varying λ and n when generating correlation profiles using the fluctuations on an arbitrarily-selected flow field for our largest (Figure II BA) and smallest (II BB) animals. As expected, increasing λ does increase the strength of correlations at larger spatial distances, but only very subtly, and the effect is more pronounced effect

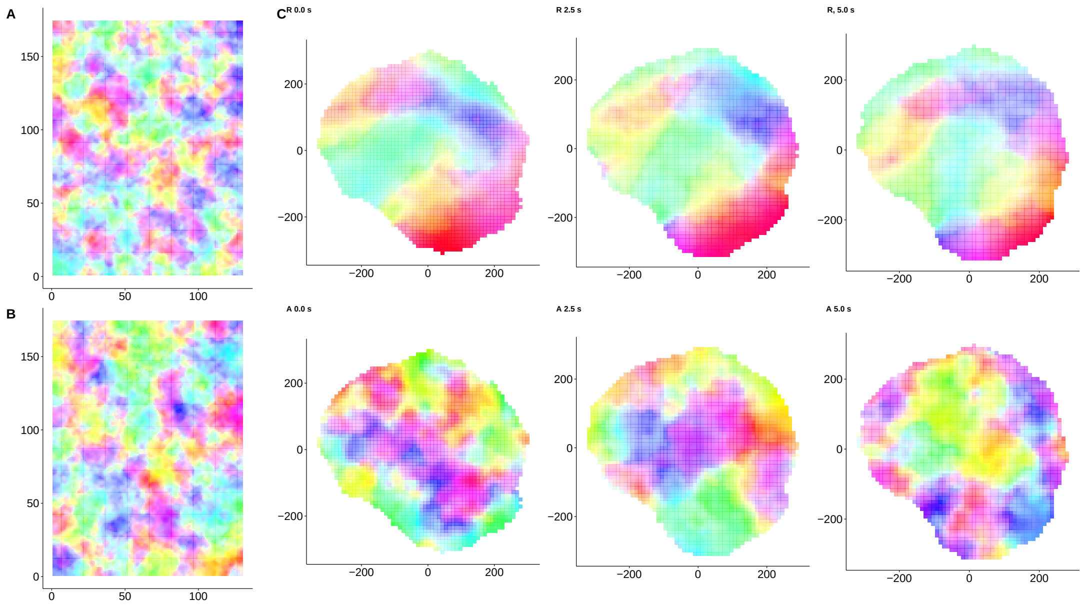

Fig. S 2. **Comparing animal velocity fluctuations to white noise.** (A) A full velocity field generate from two subsequent scrambled images. (B) The velocity field generated for the subsequent scrambled image. (C) Time series comparison of velocity fluctuations measured from the animal's movement (top) with those generated by scrambled images (bottom) at 0, 2.5, and 5 seconds of recording.

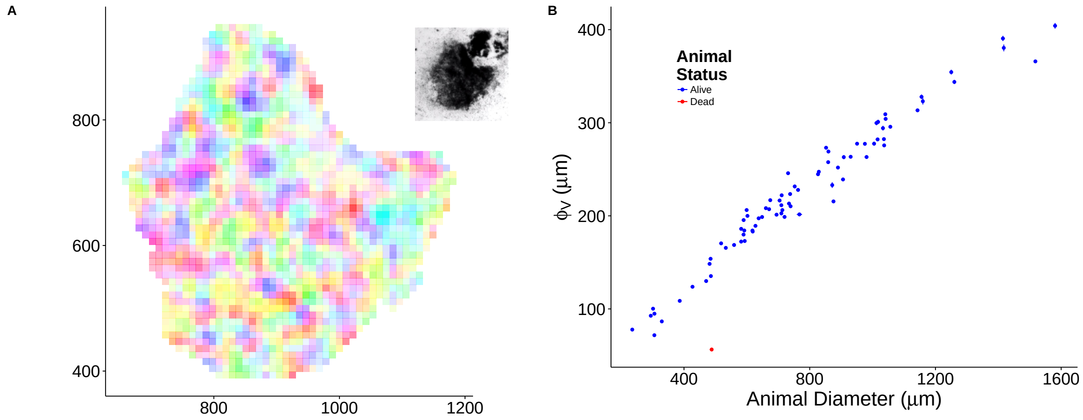

Fig. S 3. **Fluctuation correlations in dead animals are smaller than all live animal measurements.** (A) A snapshot of the velocity fluctuation field for a dead animal, inset: image of the dead animal. Axes are in micrometers. (B) Comparison of the correlation length of velocity fluctuations from a dead animal in relation to the live measurements. The correlation length is smaller than that recorded for even our smallest live specimens.

sin smaller animals. This occurs when λ is set to extremely large values that approach the size of the animal, such that the motion at any point within the animal is estimated by the movement of a substantial fraction or all of the animal. Though our results vary with the parameters used, we find that such variation is insufficient to explain the strength of the scaling phenomena we observe on susceptibility χ. Though χ increases by up to 28 percent in our smallest animals and up to 20 percent in our largest animals as we vary λ from 15 to 240 (II BC), these increases occur in near-proportion to each other when one uses reasonable values of λ that are below 100 pixels. In this case, the ratio of these two susceptibility values, $\frac{\chi_L}{\chi_S}$, changes by less than 10 percent (II BD), which is insufficient to explain the much smaller value of β for the fitted sublinear scaling of this observable in our organisms.

In order to establish ground truth regarding our measurement error of these correlations using the optical flow parameters and our isolation of fluctuations by subtraction of collective modes, we produced synthetic videos of textures deforming with a known correlation length. We simulated particles whose movement is determined by a random velocity $\mathbf{v}_i$. We then use a two-dimensional Gaussian kernel smoothing function with varying kernel sizes

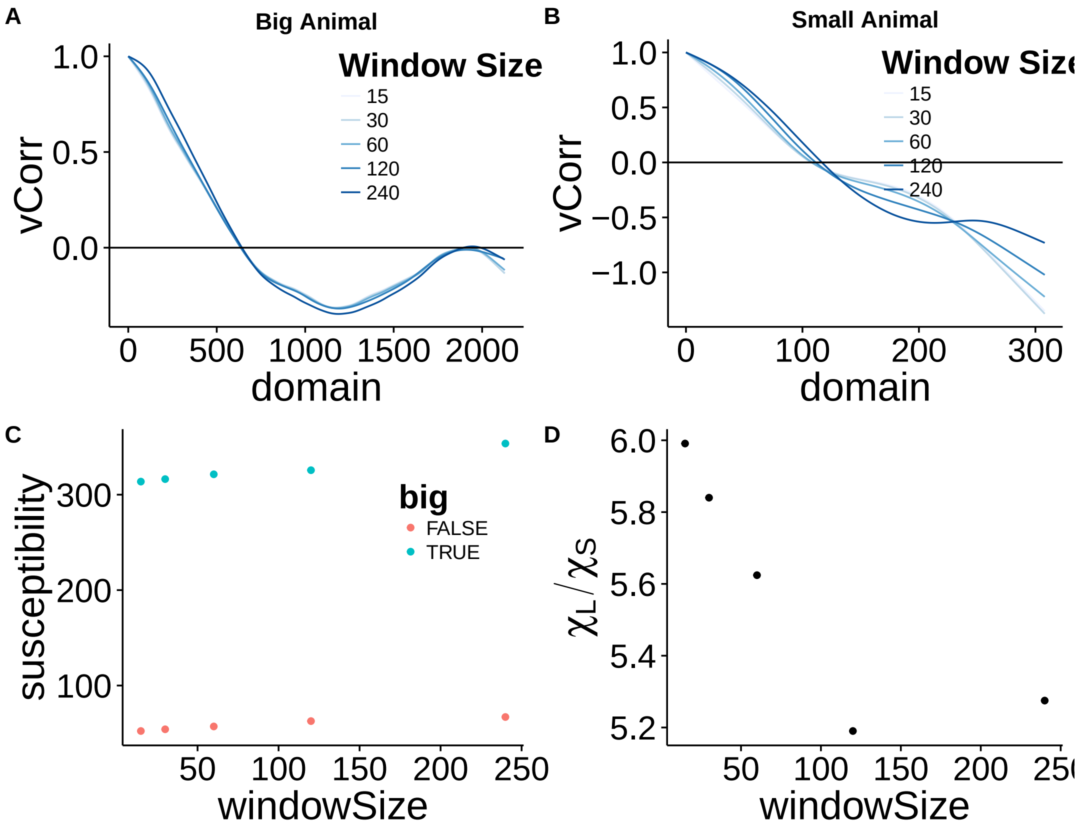

Fig. S 4. **Effect of window size on correlation profiles**. The correlation profiles generated for two randomly-selected frames from our smallest (A) and largest (B) animal specimens, measured using the fluctuations of generated vector fields with different values of λ. (C) χ for the small and large animals at varying values of λ. (D) The ratio of susceptibility of the large animal in comparison with the smaller animal.

k to correlate the velocities of particles across different spatial distances. Calculating the correlations among the particles based on their positions and velocities allows us to establish a ground truth to the movement's correlation structure. We test the output of our optical flow algorithm against this ground truth by making an image sequence of the particles visualized as large dots that have their positions in each frame updated according to their respective velocities. This produces a deforming random texture with a known correlation structure. We ran our optical flow algorithm on these videos and by varying λ produce flow fields with different levels of granularity and smoothness. Figure II BA, provides a snapshot of the original image, with figure II BB showing the velocity fluctuation of each particle and II BC showing the estimated velocity field of these moving particles using optical flow.

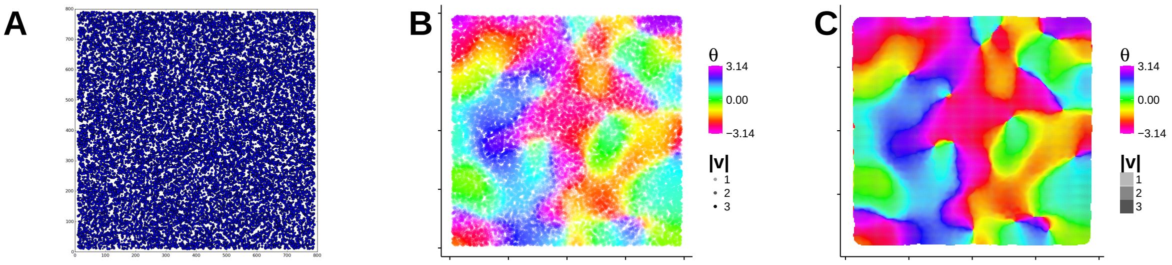

Fig. S 5. **Optical flow reconstruction of particle simulation.** (A) Representative image of a deformable composed particles moving with correlated velocities. (B) The velocity fluctuations of all of the particles. (C) The velocity field measured on a video of moving particles ($\lambda = 45, k = 51$).

We can compare the correlation profiles calculated from velocity fields generated by measuring optical flow in

videos made of plots of our moving particles, $C(r)_f$, and compare these correlation profiles $C(r)_p$ to the ground truth correlation profile based on the actual particle positions and velocities . The results of this analysis are shown in figure II B. As is expected, the correlated domain size increases with the size of Gaussian kernel smoother k (thick black line). When we compare this ground truth correlation profile to the $C(r)_f$ profiles produced with varying values of λ, we find that $C(r)_{f(k,\lambda)}$ closely follows $C(r)_{p(k)}$ provided that λ is not excessively greater than k. $C(r)_{f(k,\lambda)}$ only grossly overestimates $C(r)_{p(k)}$ when λ is approximately an order of magnitude greater than k.

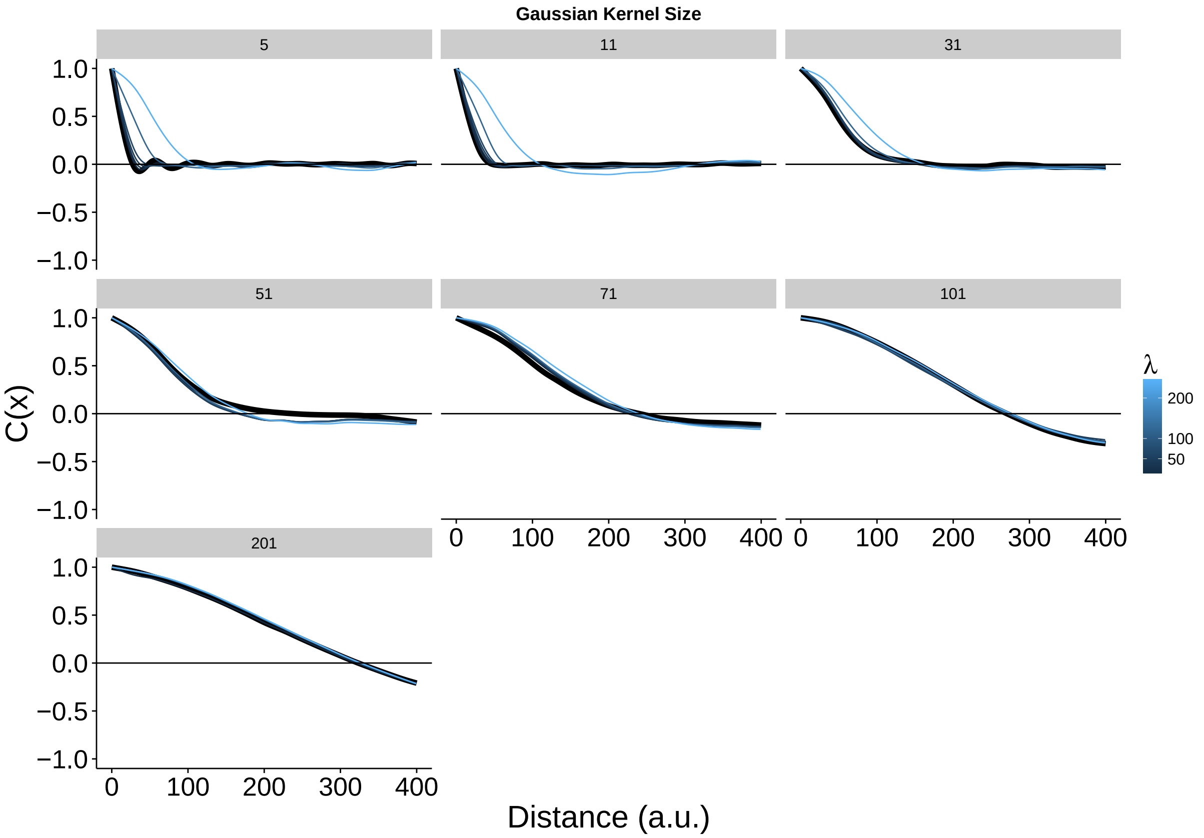

Fig. S 6. **Effect of changing the averaging window size of optical flow on the correlation profiles**. Velocity correlation profiles measured with exact particle positions and velocities (solid black line) and estimates of this correlation function by using optical flow on videos of these moving particles. The Gaussian smoother kernel size has a direct effect on the actual correlation length and strength among particles. For each kernel size, optical flow was performed on videos, varying the averaging window size (λ).

We quantify this effect of λ on the error in the correlation profile estimation for any given $C(r)_{p(k)}$ by comparing the ratio of the the cumulative correlation (integral) of the correlation profiles computed from particles and from the flow field estimation. We find that error is induced in the flow estimate only when particles are correlated over a relatively short range by using a small k (Figure II BA), and this occurs only when the the averaging window λ far exceeds the actual value of k (II BB). Unfortunately the inability to track cells within an actual animal precludes a direct estimation of k. However, k has a systematic effect on the observed correlation length ϕ for a system. We can therefore consider how the error in estimating χ is affected by the ratio of λ utilized in flow estimation with the observed ϕ. We find that λ only induces error in our measurement when it grossly exceeds the observed ϕ (Figure II BC). When we consider the range of $\frac{\lambda}{\phi}$ for our animals, we find that at most the smallest animals may have a slightly overestimated internal correlations, with the largest animals being unaffected by our chosen value of k. Therefore, the sub-linear scaling of χ with animal size is at best underreported in our current results, whereby a more accurate direct measure of cell movement could only produce an even more exaggerated sub-linearity.

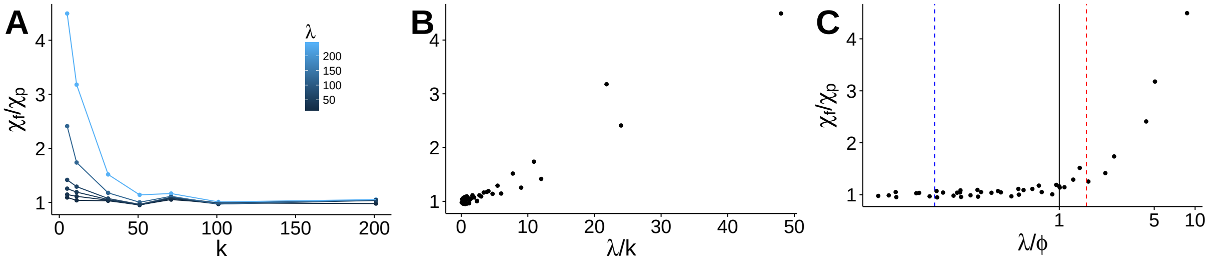

Fig. S 7. **Only overestimation of correlations is possible with optical flow, and occurs only when the smoothing window exceeds the actual correlation length in a system.** (A) χ_f only diverges from χ_p by overestimation, and only for when correlations are much shorter than the flow averaging window size. (B) Overestimation dependence on the ratio between the flow estimation window size and the actual correlation kernel size. (C) Dependence of the in the cumulative correlation estimation on the ratio between the averaging window size and the observed correlation length (blue line: ratio of ϕ_v for the largest animal relative to the averaging window used in empirical flow fields; red line: similar ratio for our smallest observed animal).

III. CORRELATION MEASUREMENT METHODS

A. Comparing velocity, direction, and speed correlations

Our main text focuses on spatial correlations in velocity, which is a combination of direction and speed. While we found linear correlations in velocity, we wanted to determine if a similar pattern held for both direction and speed separately. By repeating our calculation on these two quantities we show that both direction (figure III AA) and speed (figure III AC) show the same monotonically decreasing profiles of correlation with distance between two cells, with profiles having a single x-intercept. Furthermore, when we plot this x-intercept against the diameter of the animal, we see the same linear scaling (figures III A B and D, respectively). This demonstrates that the linear scaling we observe is not specific to velocity, but holds for a variety of quantities that describe the movement of cells within the animal.

B. Sensitivity to averaging methods: ensemble *vs.* spatial averaging

A key problem in statistical mechanics of active matter is understanding how to perform the averaging necessary to define statistical quantities. Many of the previous studies that have looked at such systems have considered only spatial averages at snapshots in time [4–7], presumably because such systems are non-stationary. However, one could equally perform an ensemble averages, combining measurements from different points in time, and determining if there are any nonlinear effects that result in an inconsistency between the two averaging methods. We compared these two methods of averaging by measuring the correlation profiles at 100 instances in time for each animal (the spatial average) and then calculating the averaging correlation profile for each animal across all instances in time (the ensemble average). We then measured the mean ϕ for the spatial average profiles, $\langle \phi(C(r), t) \rangle$, and compared it to the ϕ value of the mean profile $\phi(\langle C(r) \rangle)$, the results shown in Figure 9A, B, and C. A similar comparison was also performed for χ (Figure 9D, E, and F). We find that the spatial and ensemble average quantities correspond well with one another for all of our correlation measurements. There is a slight but systematic overestimate of χ when computed using the spatial method, but it is a very minor effect present at all animal sizes and therefore is unlikely to be the cause of the sub-linear scaling phenomenon that we report in the main text.

IV. EFFECT OF SIZE ON ORDER MEASURES

One concern of using a unified order parameter O, as we have done in the main text, is whether the effect on size on internal coordination is a reflection of the size effect on internal order in the component factors, or if it is an artifact of the way in which our order parameters are aggregated into this unified parameter. We therefore measured the effect of size on the mean order for both the unified order, as well as for polarization, rotation, and dilatation. We find that size has a robust, diminishing effect on order both for the unified parameter ($t = -5.7; p < 10^-6$), polarization ($t = -3.37, p = 0.001$), and rotation (t = -2.55, p = 0.01). The dilatation order parameter does not appear to be significant at any animal size, being close to zero, and therefore no effect of size on this factor can

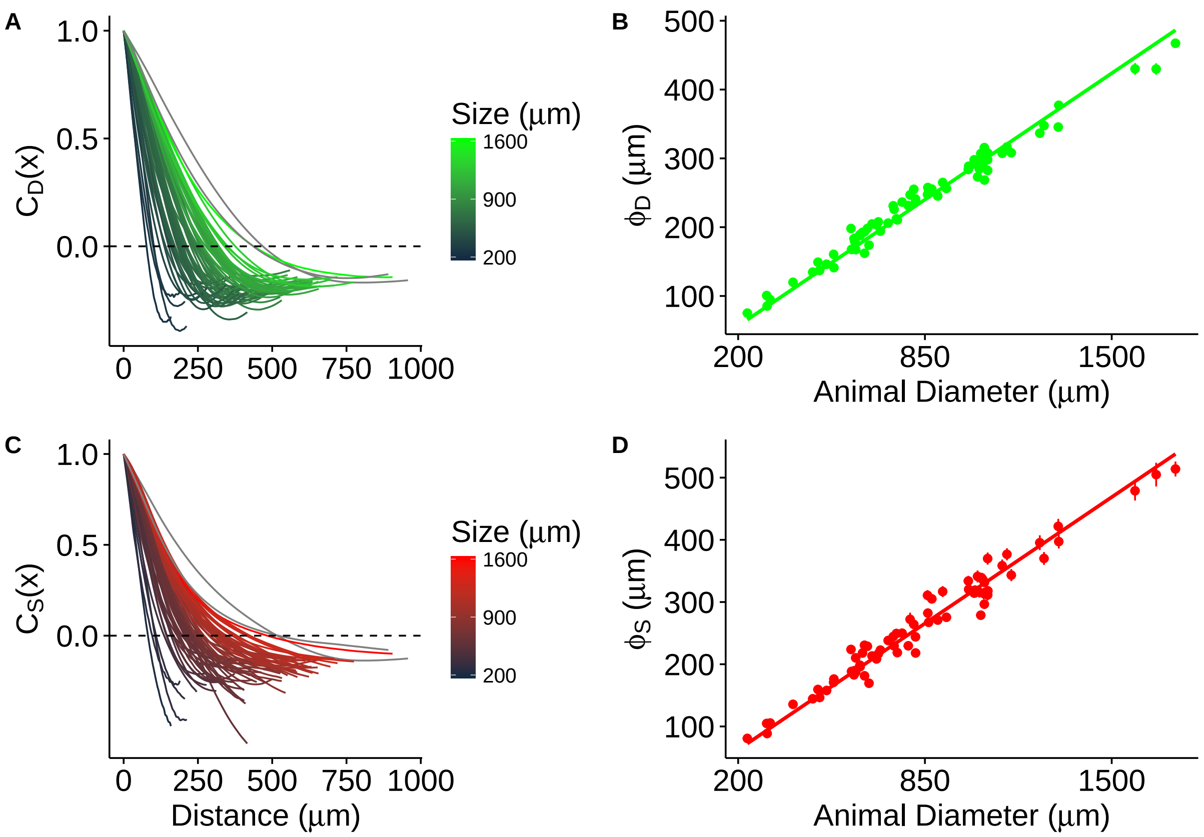

Fig. S 8. **Scale-free correlations are present in both direction and speed of cellular movement in *T. adhaerens*.** (A) Correlation profiles in movement direction between cells in different *T. adhaerens* individuals. Brigher color represents larger animals. (B) The x-intercept of profiles the direction correlation profiles, showing a linear correlation with animal size. (C) Correlation profiles in instantaneous speed of cells in different *T. adharens* indvidual s. Brighter colors represent largeranimals

be detected ($t = 1.48, p = 0.14$). We are therefore confident that the effect of size on the unified order parameter accurately reflects a similar effect on the constitutive order measures, and is not an artifact of our calculation.

V. NON-SIGNIFICANT FACTORS ON CORRELATION STRUCTURE

Network topology is an important factor in information propagation. In sheet-like systems such as Placozoa, changes in network topology can only be achieved by changes in shape, which cause . We noted that larger Placozoa have more irregular shapes, deviating substantially from the disk morphology of smaller specimens (Figure 11A). We quantify this deviation using circularity, $C = \frac{4\pi A}{P^2}$ (Figure 11A), and determined the relationship of this factor with the dimensionless quantity $\frac{\theta}{L}$. Though there may initially appear to be a slight correlation between the circularity of an animal and this ration (Figure 11B), we find that this relationship is statistically not significant when we account for the effect of L itself on $\frac{\phi}{L}$ (linear regression: $\frac{\phi}{L} = \beta_1 L + \beta_2 C + \gamma; p(\beta_2) = 0.71$).

VI. THE RELATION BETWEEN COLLECTIVE ORDER AND LOCOMOTION

An important consideration is whether the degree of collective order is consequential for an animal's fitness, and one proximal assessment of this is whether collective order and displacement through locomotion are related. In order to understand this relationship, we compared the centroid trajectory for each animal with its instantaneous collective order (polarization). We randomly sampled 500 time points in each animal's trajectory, measuring the collective order at time t and the resultant displacement between $t - 5$ and $t + 5$ seconds.

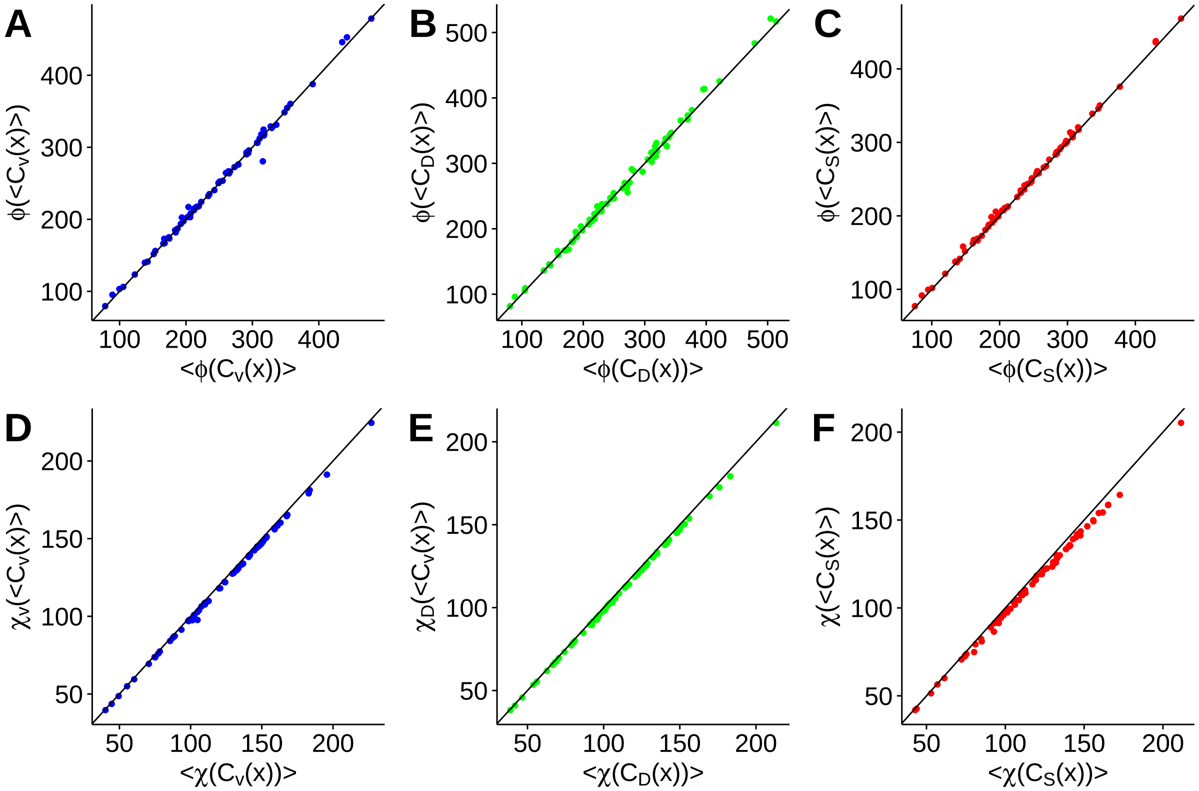

Fig. S 9. **Comparison of averaging methods.** In all panels the black line represents $y = x$, the spatial average is plotted on the x-axis and the ensemble average is plotted on the y-axis. The comparison for the correlation lengths ϕ (top) and susceptibility χ (bottom) are shown for three different type of correlations, from left to right: velocity (A, D), direction, (B, E), and speed (C, F). In all cases $\mathrm{R}^2 > 0.99$.

We find that an animal's centroid displacement is significantly correlated with an animal's collective order, with a mean correlation (per animal) of $\bar{\rho} = 0.65$, and a standard deviation of $\sigma_\rho = 0.14$. This suggests that collective order is reflective of an animal's locomotion effectiveness, and therefore will have an impact on animal fitness under natural conditions.

VII. COLLECTIVE ORDER

The ordered forms of collective movement within *T. adhaerens* individuals varies substantially and systematically with animal size. In our study we consider a collective mode of movement to be any transformation in the spatial position of a collective's components that can be explain by an affine transform, or a combination of rotation, translation, and dilatation. These transformations are defined in the Methods section of the main text. We present Figure 16A to illustrate the dynamics of these order parameters for a representative small animal, with Figure 16B showing the distribution for these three measures throughout the entire recording. As is typically for such a small animal, the dynamic range of dilatational order is quite small when compared to the rotational and polarization order, emphasizing the solid-body like motion of such animals. When we compare our largest and smallest animals (Figure 16C), we find that the behavior of the smallest individual is well-represented by a thin manifold defining a mixture of behaviors ranging from high polarization to high rotation. Our largest individual exhibits much more disordered locomotion, with a greater proportion of time spent in lower rotation and polarization states. We can consider a general value of collective order, defined as $O = \sqrt{P^2 + R^2 + \Lambda^2}$, we find that O decreases significantly with increasing animal size. Values of O from five animals - sampled uniformly across the range of animal sizes - illustrates this decrease in order with increasing size (Figure 16D).

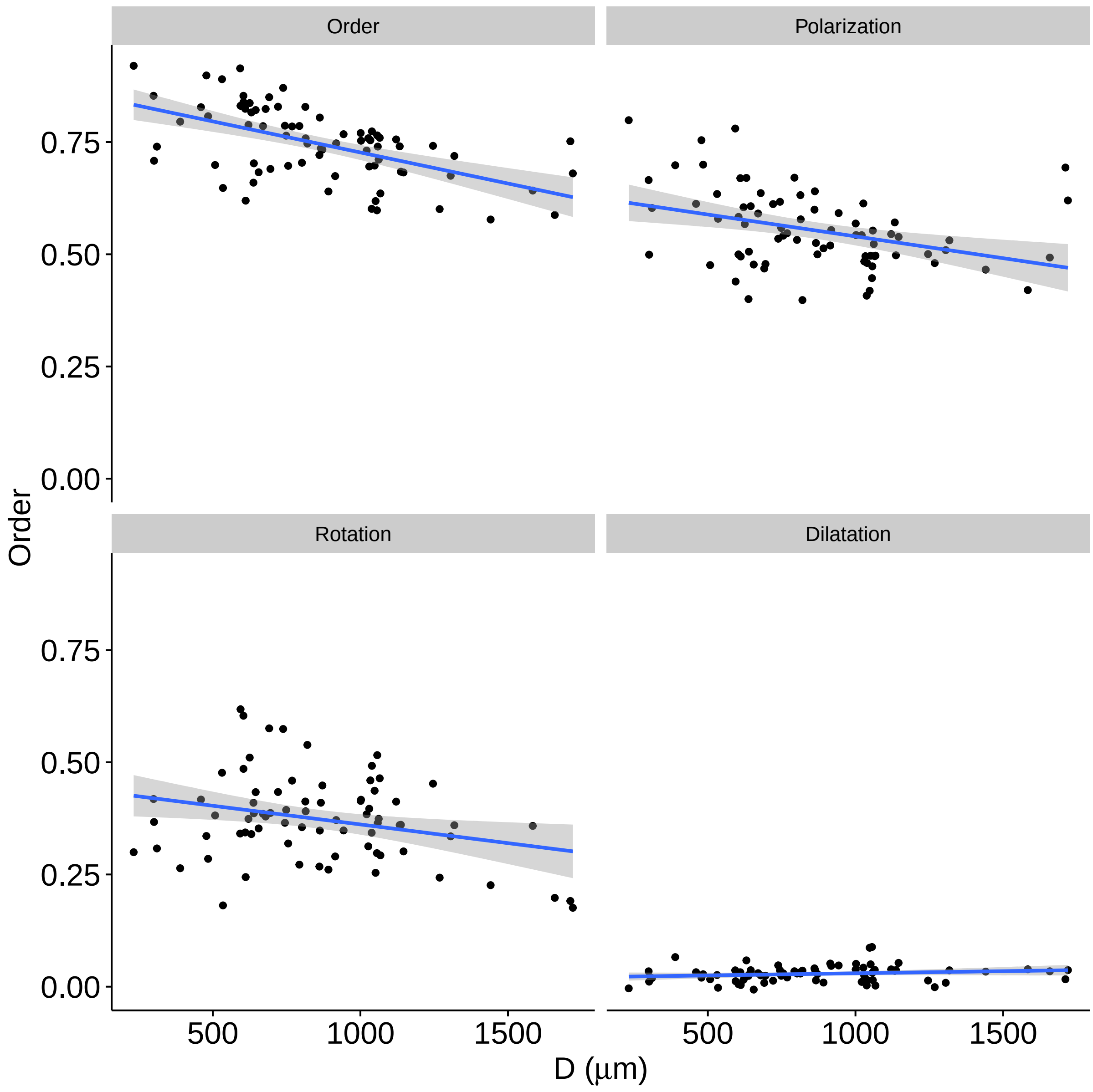

Fig. S 10. **The effect of size on unified and constituent order parameters**.

VIII. CELL SIZE IS INVARIANT TO ANIMAL SIZE

Our arguments about the effect of size on correlation structure require that cell count increase in proportion with animal area. No data on the ultrastructure of *T. adhaerens* indicate any change in cell size with animal size [8–10]. We nevertheless tested against this assumption by measuring the density of epithelial cells in animals of varying size. We took measured the diameter of cells within images of an animal's lower epithelium taken at 200x magnification under bright field conditions using an inverted microscope. Cells are roughly visible as dark spots that dot the animal tissue, though it is difficult to identify exact cell boundaries without staining techniques. We used ImageJ to perform histogram equalization for each image and then use the built-in local minimum detection algorithm in ImageJ to count the number of minima within a selected sub-region of the animal. We do this in several regions from several arbitrarily-selected frames that we recorded for each animal. The tolerance for detecting local minima was 5 intensity values in an image of 8-bit color depth. As can be seen in Figure 13, though there is substantial variability in cell density for a single animal, reflecting an animal's propensity to stretch or contract, there is no systematic effect on size on cell density. Therefore, we assume cell count increases linearly with animal area in our experiments.

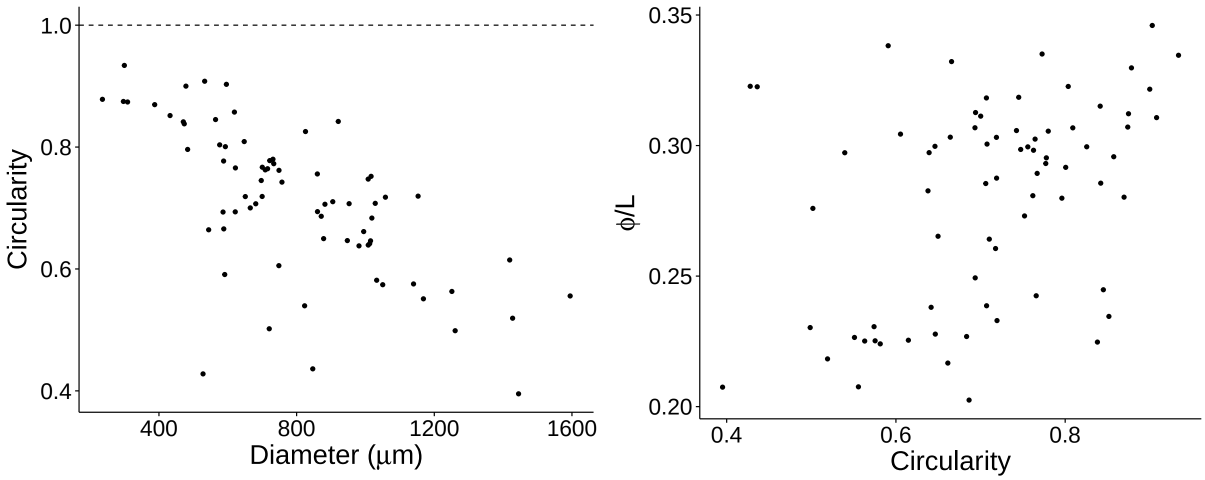

Fig. S 11. **Effect of shape on correlation structure.** (A) Deviation from perfect circularity with increasing size (dashed line: perfect circle). (B) Circularity effect on proportional correlation length extent

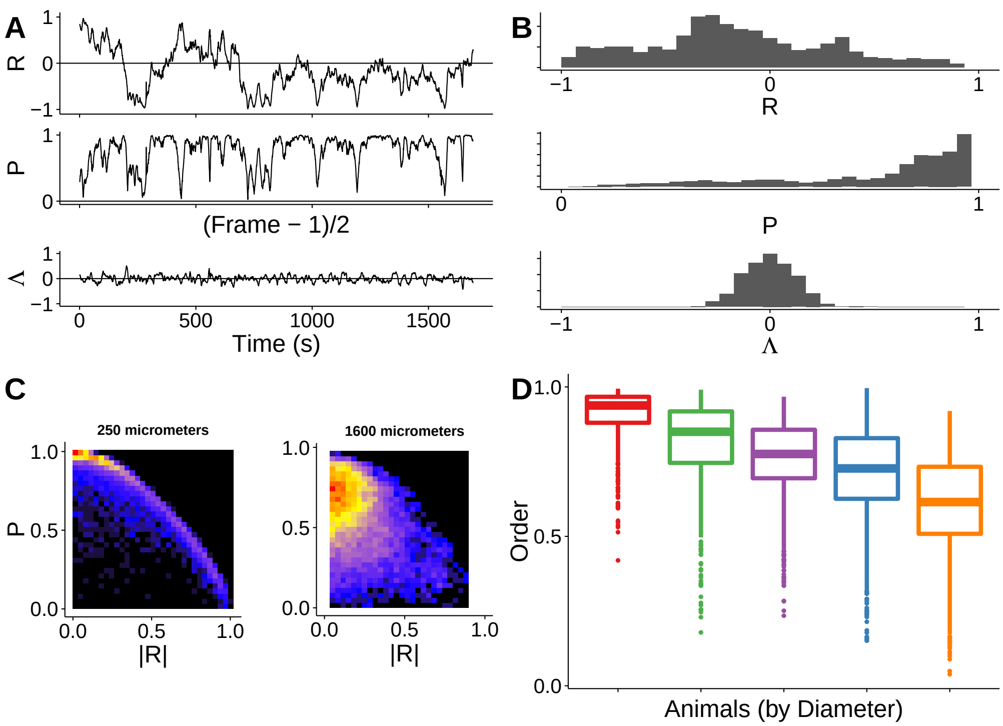

Fig. S 12. **Collective modes of locomotion in *T. adhaerens*.** (A) A representative time series of the rotational (R), polarized (P), and dilatational (Λ) modes of collective order, showing large and high frequency variability in all parameters. (B) Histograms of the observed values for all three order parameters throughout the entire recording for one arbitrarily-selected animal. (C) Phase space histograms of the collective rotation and polarization order for the smallest and largest animals. (D) The collective order for five animals, ranked by their mean size.

IX. NUMERICAL MODEL

The dynamics of collective movement of cells within animals will is the result of multiple inter- and intracellular mechanical forces, and possibly chemical signaling between neighboring cells. In spite of recent advances in uncovering the detailed structure of *T. adherens* [8], the dynamics of cell coordination remain unknown. In order to systematically study these dynamics, we use simplified model description, based on three main assumptions:

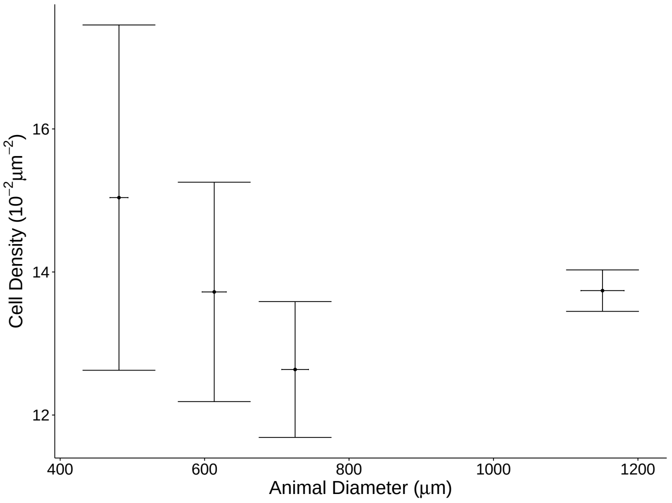

Fig. S 13. **Cell density is invariant with cell size.** Cell counts were measured in 15 sub-regions of the animal for each animal, with regions ranging ins size from 2000 to 5000 μm^2. Error bars represent standard deviation.

- interactions between parts of the animal are local, restricted only to the first shell of neighboring units (Voronoi neighborhood),
- the mechanical interactions can be mapped to an effective "mechanical" spring-like forces
- each unit is self-propelled with a preferred direction of motion, which on a finite time scale relaxes towards the average direction of the resulting mechanical force. Furthermore the heading direction of self-propulsion is subject to fluctuations.

We model individual Placozoa as a two-dimensional ensemble of coupled self-propelled particles (SPP). Each particle corresponds to a cellular 'disc' 10 μm in diameter, the maximum resolved scale that allows us to parametrize our simulations from experimental data. The dynamics of such an ensemble of N particles is described by a set of stochastic differential equations. The motion of each particle i ($i = 1, \ldots, N$) in 2D is described by the overdamped equations of motion given by Equation 12 in the main text.

Each particle interacts with its Voronoi neighbors via linear springs with the equilibrium distance r_0 set by the particle diameter and the spring constant μ. Thus, a particle i is repelled from a neighbor j for $r_{ji} < r_0$ and attracted for $r_{ji} > r_0$. Here, $r_{ji} = |\vec{r}_{ji}|$, with $\vec{r}_{ji} = \vec{r}_j - \vec{r}_i$. The total force acting on an agent is obtained from summing up the contribution from its neighbors:

$$\vec{F}_i = \sum_{j \in neighb} \mu(r_{ji} - r_0)\hat{r}_{ji} \tag{1}$$

with $\hat{r}_{ji} = \vec{r}_{ji}/r_{ji}$.

Regarding the interaction neighborhood, we can distinguish fundamentally between two model variants:

1. static: the Voronoi network is fixed after an initial relaxation period, so that particle maintain their neighbors
2. dynamic: the interaction network is recalculated at each step, so that particles may change positions within the network.

Here we confirm that the results from the static network model, presented in the main text, are generalizable to the dynamic neighborhood model, shown in Figure 14. In such simulations, particles occasionally intercalate and switch neighbors due to fluctuations in the collective density and particle heading. In spite of this intercalation and neighbor-switching, which does not occur in the fixed network simulations, we note the same relation between the effective noise η^* and order (A), with the relationship being more strongly negative in larger animals (B). As in our fixed network simulations, the correlation length θ_v increases effectively linearly with the diameter of the collective (C), while the susceptibility χ_v increases in the characteristic sub-linear fashion reported in the main text. That our fixed network simulation results are recapitulate here suggests that the size-order trade-off in *T. adhaerens* is a result of the elastic interactions with Voronoi neighbors independent on neighbor switching. It should be noted that

our dynamic simulations are also in agreement with observations in other collectively moving systems with dynamic interaction networks, such as flocks of birds, where it was also shown that neighbor-switching plays only a secondary role in information propagation [11] due to the timescale of neighbor-switching, which is slow relative to directional equilibration among neighboring agents.

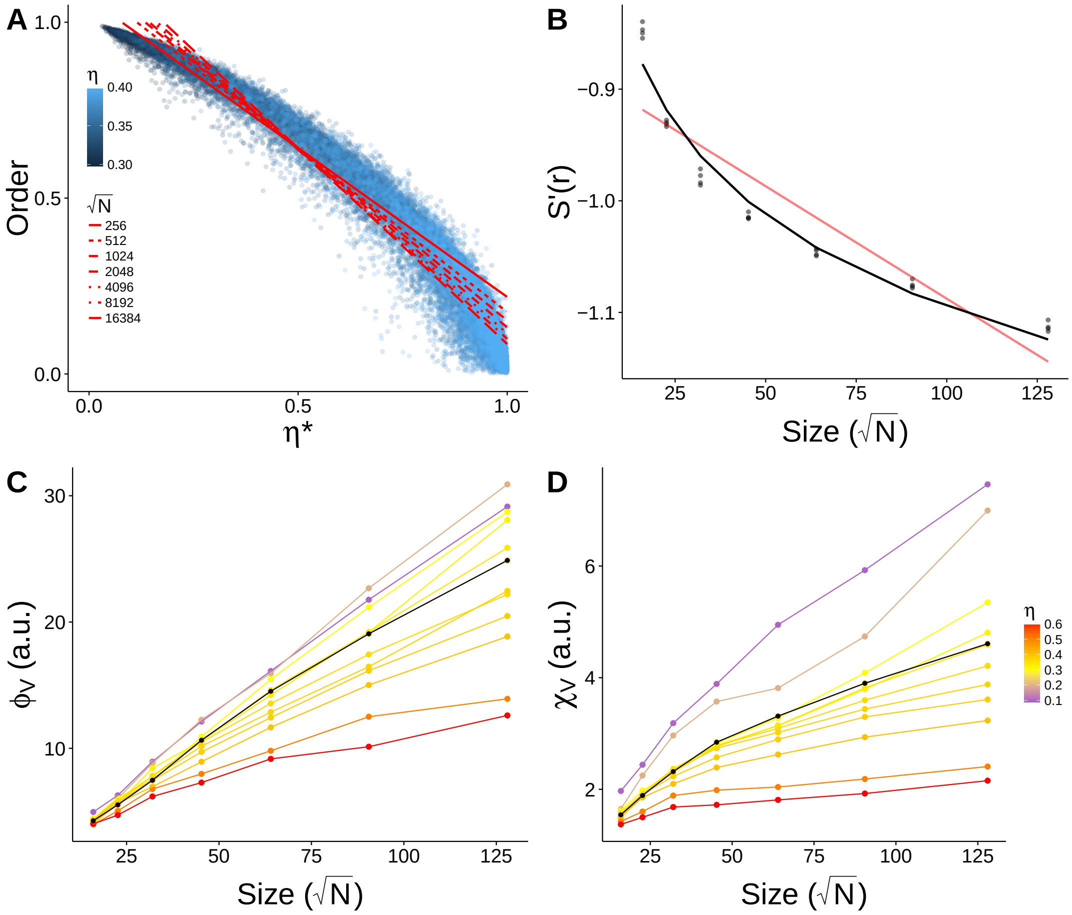

Fig. S 14. **Scaling properties of simulated sheets with dynamic interaction networks**. (A) The relation between instantaneous fluctuation energy fraction and collective order for system of different size for systems with η ranging from 0.3 to 0.4. (B) The steepness of the phase transition (derivative of panel A) for systems of varying size. (C) The effect of size and η on the correlation length and on susceptibility (D).

X. DETERMINING η_c USING THE BINDER CUMULANT

W by using the the Binder cumulant $U = 1 - \frac{\langle \mathbf{v}^4 \rangle}{3\langle \mathbf{v}^2 \rangle^2}$, where $\mathbf{v}$ is the ensemble average velocity for each system. By plotting U as we systematically vary η for various system sizes L, and estimate η_C from the common intersection point of these curves [12].

XI. THE EFFECT OF THE NOISE CONTROL PARAMETER ON FLUCTUATION ENERGY

One limitation of empirical data from *T. adhaerens* is that we cannot directly measure the control parameters that affect its behavior, such as the alignment noise η that affects the behavior of our simulations, and must instead infer this parameter from the fraction of kinetic energy that is channeled into velocity fluctuations, $\eta^* = \frac{\langle |\mathbf{u}^2|| \rangle}{\langle |\mathbf{u}^2|| \rangle}$. From our simulations, we determined that η^* increases monotonically and in a sigmoidal fashion with η (Figure 15A). However,

in the critical regime, where the order-disorder phase transition occurs, the relationship between these two quantities is effectively linear (B). Therefore, η^* is an accurate proxy for η in near-critical systems such as *T. adhaerens* .

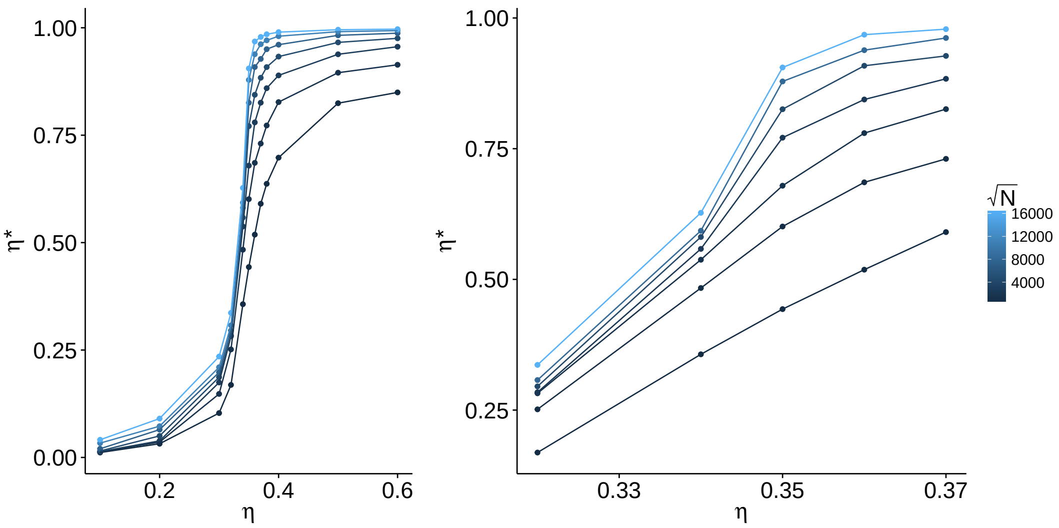

Fig. S 15. **The relation between the noise control parameter and effective noise.** (A) Effective noise increases in a sigmoidal fashion with the noise control parameter. (B) In the critical regime, the relationship between noise and effective noise is approximately linear.

XII. RESPONSE OF CORRELATION PROFILES TO VARYING INTRINSIC NOISE IN SIMULATIONS

We identify two scaling phenomena We systematically varied the intrinsic angular noise in our simulations to determine if our results are sensitive to this parameter, repeating our correlation measures under these different noise regimes. The correlation profiles all show the same characteristic increasing concave decay with increasing system size (Figure 16) When we plot the respective susceptibility of these profiles, we observe a sub-linear trend of susceptibility with system size at all noise levels, with the exception of when noise is extremely low (Figure 17).

XIII. ALTERNATIVE METHODS OF RESCALING

Rescaling r by the correlation length ϕ is one possible method of analyzing the size invariance of correlation structure, and is the method used in other studies of a similar nature [4, 7, 13]. Nevertheless, the poor collapse of the correlation profiles in our system when using this method prompted us to consider other methods by which we could produce rescaled correlation profiles. One such method is χ^2 minimization, where the domain of each profile is rescaled a scalar parameter α, selected in order to minimize the sum of the squared difference between the two profiles, divided by the uncertainty [14]. The results of such a minimization are shown in Figure XIII. The correlation profiles again do not collapse on top of each other in an invariant fashion. By observing the concavity and convexity of each profile, it is trivial to conclude that no better rescaling is possible.

---

[1] B. K. Horn and B. G. Schunck, Artificial Intelligence **17**, 185 (1981).
[2] G. Farnebäck, in *Image Analysis*, Vol. 2749, edited by G. Goos, J. Hartmanis, J. van Leeuwen, J. Bigun, and T. Gustavsson (Springer Berlin Heidelberg, Berlin, Heidelberg, 2003) pp. 363–370, dOI: 10.1007/3-540-45103-X_50.
[3] S. Leblanc, "FishFlow," (2017).
[4] A. Cavagna, A. Cimarelli, I. Giardina, G. Parisi, R. Santagati, F. Stefanini, and M. Viale, Proceedings of the National Academy of Sciences **107**, 11865 (2010).
[5] A. Attanasi, A. Cavagna, L. Del Castello, I. Giardina, A. Jelic, S. Melillo, L. Parisi, O. Pohl, E. Shen, and M. Viale, Journal of The Royal Society Interface **12**, 20150319 (2015).

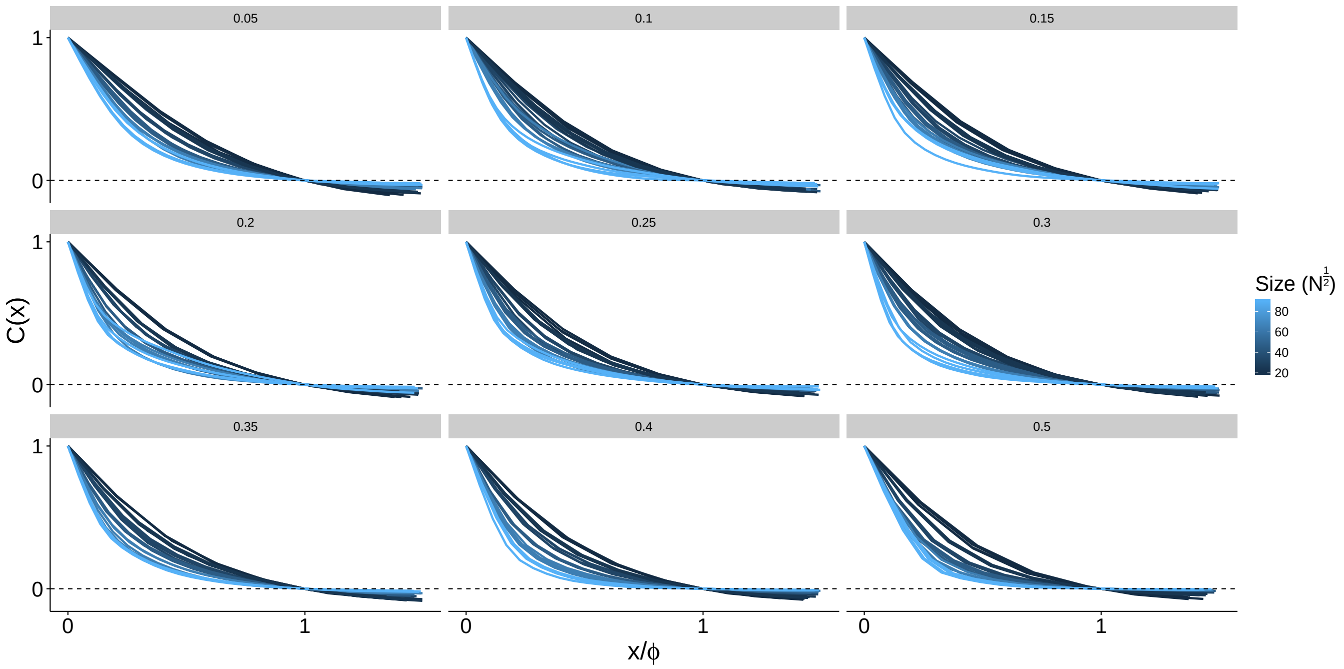

Fig. S 16. **Size-mediated effects on correlation strength are robust to noise.** The correlation profiles for simulated systems of different sizes, with distances rescaled by their respective correlation lengths. We see that under a variety of conditions

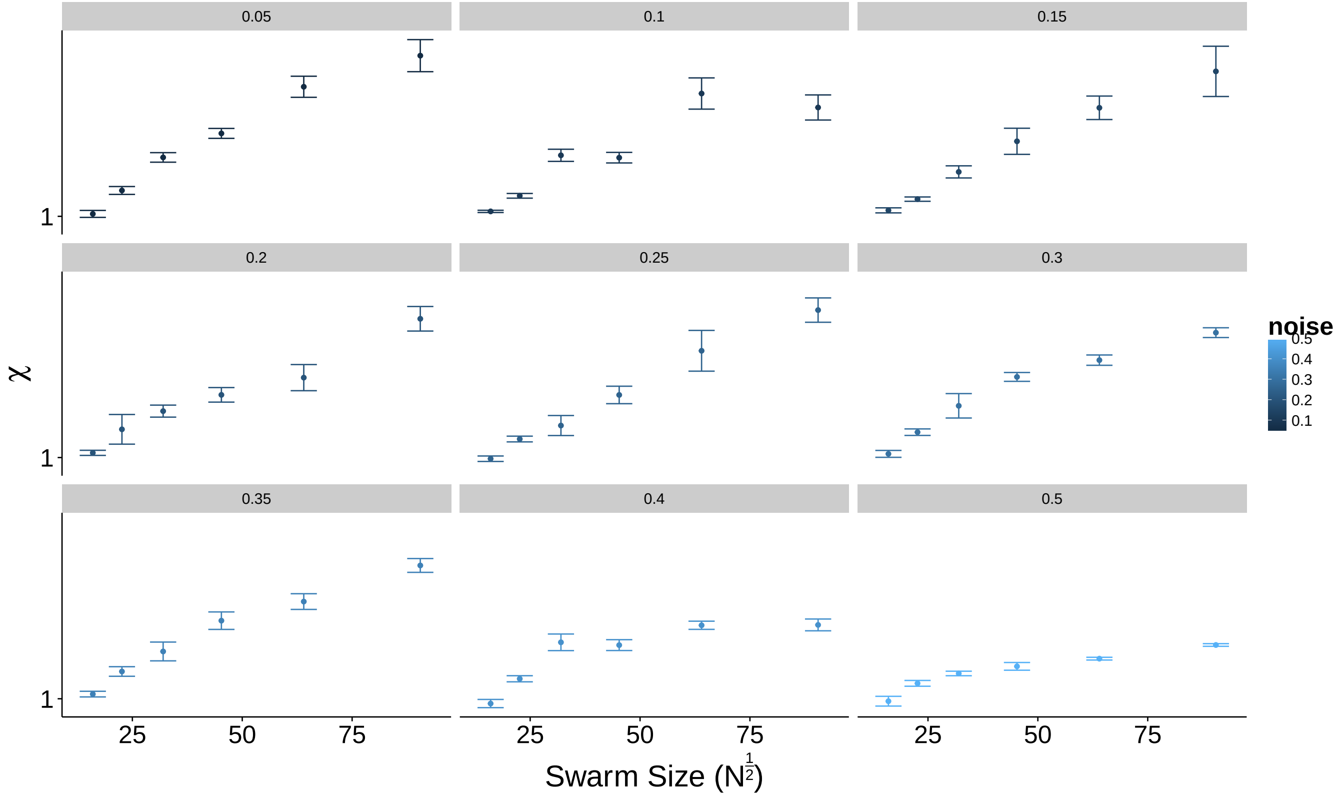

Fig. S 17. Susceptibility increases sub-linearly with system size for all noise levels, though at low noise this trend approaches linearity.

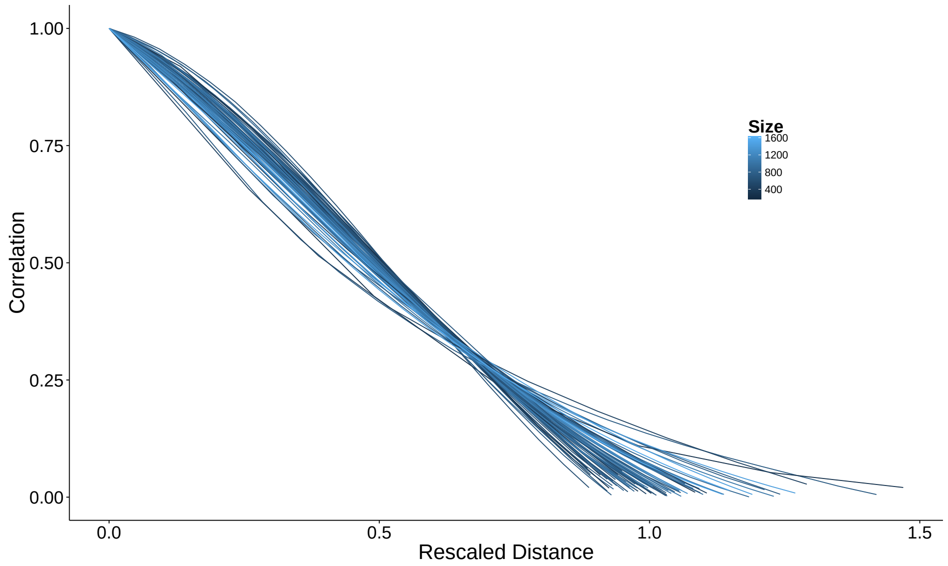

Fig. S 18. **χ-square minimization does not result in size invariance.** The correlation profiles of all *T. adhaerens* individuals in our dataset, rescaled to align with the profile from the smallest individual after its domain was normalized by its correlation length (zero-intercept).

[6] C. Huepe, E. Ferrante, T. Wenseleers, and A. E. Turgut, Journal of Statistical Physics **158**, 549 (2015).

[7] A. Chakraborty and K. Bhattacharya, EPL (Europhysics Letters) **116**, 48001 (2016).

[8] C. L. Smith, F. Varoqueaux, M. Kittelmann, R. N. Azzam, B. Cooper, C. A. Winters, M. Eitel, D. Fasshauer, and T. S. Reese, Current Biology **24**, 1565 (2014).

[9] L. Guidi, M. Eitel, E. Cesarini, B. Schierwater, and M. Balsamo, Journal of Morphology **272**, 371 (2011).

[10] K. G. Grell and A. Ruthmann, in *Microscopy Anatomy of Invertebrates*, Vol. 2, edited by F. W. Harrison (Wiley-Liss, New York, 1991) pp. 13–27.

[11] T. Mora, A. M. Walczak, L. Del Castello, F. Ginelli, S. Melillo, L. Parisi, M. Viale, A. Cavagna, and I. Giardina, Nature Physics (2016), 10.1038/nphys3846.

[12] K. Binder and D. W. Heerman, *Monte Carlo Simulations in Statistical Physics. An Introduction*, 1st ed., Springer series in solid-state sciences No. 80 (Springer, Berlin, Germany, 1988).

[13] X. Chen, X. Dong, A. Be'er, H. L. Swinney, and H. P. Zhang, Physical Review Letters **108** (2012), 10.1103/PhysRevLett.108.148101.

[14] S. L. Meyer, *Data analysis for scientists and engineers* (Peer Management Consultants, Ltd., Evanston, IL, 1992) oCLC: 42795291.
